# RNA-Seq Transcriptome Profiling Identifies *CRISPLD2* as a Glucocorticoid Responsive Gene that Modulates Cytokine Function in Airway Smooth Muscle Cells

**DOI:** 10.1371/journal.pone.0099625

**Published:** 2014-06-13

**Authors:** Blanca E. Himes, Xiaofeng Jiang, Peter Wagner, Ruoxi Hu, Qiyu Wang, Barbara Klanderman, Reid M. Whitaker, Qingling Duan, Jessica Lasky-Su, Christina Nikolos, William Jester, Martin Johnson, Reynold A. Panettieri, Kelan G. Tantisira, Scott T. Weiss, Quan Lu

**Affiliations:** 1 Channing Division of Network Medicine, Brigham and Women's Hospital and Harvard Medical School, Boston, Massachusetts, United States of America; 2 Partners HealthCare Personalized Medicine, Boston, Massachusetts, United States of America; 3 Children's Hospital Informatics Program, Boston, Massachusetts, United States of America; 4 Program in Molecular and Integrative Physiological Sciences, Departments of Environmental Health, and Genetics and Complex Diseases, Harvard School of Public Health, Boston, Massachusetts, United States of America; 5 Pulmonary, Allergy and Critical Care Division, University of Pennsylvania, Philadelphia, Pennsylvania, United States of America; University of Ulm, Germany

## Abstract

Asthma is a chronic inflammatory respiratory disease that affects over 300 million people worldwide. Glucocorticoids are a mainstay therapy for asthma because they exert anti-inflammatory effects in multiple lung tissues, including the airway smooth muscle (ASM). However, the mechanism by which glucocorticoids suppress inflammation in ASM remains poorly understood. Using RNA-Seq, a high-throughput sequencing method, we characterized transcriptomic changes in four primary human ASM cell lines that were treated with dexamethasone—a potent synthetic glucocorticoid (1 µM for 18 hours). Based on a Benjamini-Hochberg corrected p-value <0.05, we identified 316 differentially expressed genes, including both well known (*DUSP1, KLF15, PER1, TSC22D3*) and less investigated (*C7, CCDC69, CRISPLD2*) glucocorticoid-responsive genes. *CRISPLD2*, which encodes a secreted protein previously implicated in lung development and endotoxin regulation, was found to have SNPs that were moderately associated with inhaled corticosteroid resistance and bronchodilator response among asthma patients in two previously conducted genome-wide association studies. Quantitative RT-PCR and Western blotting showed that dexamethasone treatment significantly increased *CRISPLD2* mRNA and protein expression in ASM cells. *CRISPLD2* expression was also induced by the inflammatory cytokine IL1β, and small interfering RNA-mediated knockdown of *CRISPLD2* further increased IL1β-induced expression of *IL6* and *IL8*. Our findings offer a comprehensive view of the effect of a glucocorticoid on the ASM transcriptome and identify *CRISPLD2* as an asthma pharmacogenetics candidate gene that regulates anti-inflammatory effects of glucocorticoids in the ASM.

## Introduction

Asthma, a chronic inflammatory respiratory disease that affects over 25 million Americans and 300 million people world-wide, is characterized by variable airflow limitation and airway hyperresponsiveness [1], [2]. Glucocorticoids (GCs) are common medications used to treat various inflammatory diseases, including asthma [3]. Inhaled corticosteroids, GC medications that act directly in the lung, are among the most common asthma controller medications and treatment of asthma patients with them leads to improved clinical outcomes, including decreased asthma symptoms and exacerbations [4]. At a cellular level, GCs act by binding to GC receptors (GRs), causing them to translocate to cell nuclei where they modulate transcription of various genes in a tissue-dependent fashion [5]. The anti-inflammatory action of GCs is partly a result of 1) GC-GR complexes stimulating anti-inflammatory genes by directly binding to DNA at glucocorticoid receptor enhancer elements, and of 2) GC-GR complexes inhibiting proinflammatory transcription factors such as nuclear factor kappa-light-chain-enhancer of activated B cells (NFκB) [6]. In addition to directly reducing inflammation, GCs have been shown to affect other asthma-related phenotypes, including bronchodilation [7], airway hyperresponsiveness [8], and airway smooth muscle (ASM) contractility [9].

Many cells and tissues are involved in asthma and are targeted by GCs, including inflammatory [10], [11], airway epithelium [12], and ASM [13]. Of these, the ASM is involved in altered airway contractility [14], a major asthma-specific trait that is assessed clinically and for research studies by measures such as bronchodilator response [15] and airway hyperresponsiveness [16]. However, compared to the other airway cells, much less is known about how GCs work specifically in the ASM to alleviate asthma. Because GCs function by activating GR to directly modulate transcriptional gene expression, a better understanding of how the ASM transcriptome responds to GCs is needed to provide mechanistic insights for improving asthma therapy. Several studies have been conducted to identify GCs-induced transcript changes in the ASM. For example, two microarray-based gene expression studies have measured the effect of GCs on ASM cells using *in vitro* models where human ASM cells were stimulated with dexamethasone or fluticasone [17], [18]. Although both were limited by the inherent biases of microarrays, these studies identified some genes involved in the ASM GC response, with one focusing on validating the function of the *KLF15* gene in airway hyperresponsiveness [17] and the other on the overlap between GC and beta-agonist response of the ASM [18].

Recent advances in sequencing technologies have made possible the comprehensive and in-depth characterization of transcriptomes via a technique known as RNA-Seq [19]–[21]. Compared to the use of microarrays, RNA-Seq is able to (1) quantify more RNA species, including non-coding and novel splice variants, (2) quantify RNA at baseline, rather than only measure fold changes across conditions, and (3) cover a wider dynamic range of signal [22]. In this study, we used RNA-Seq to comprehensively characterize changes of the ASM transcriptome in response to GCs using an *in vitro* model. We identified 316 significantly differentially expressed genes representing various functional categories such as glycoprotein/extracellular matrix, vasculature and lung development, regulation of cell migration, and extracellular matrix organization. One of these genes, cysteine-rich secretory protein LCCL domain-containing, 2 (*CRISPLD2*; OMIM 612434), had single nucleotide polymorphisms (SNPs) that were nominally associated with two asthma drug response phenotypes (i.e., inhaled corticosteroid response and short-acting bronchodilator response). Functional experiments showed that in ASM cells, *CRISPLD2* mRNA and protein levels changed in response to treatment with a glucocorticoid or proinflammatory cytokine, and that knockdown of *CRISPLD2* resulted in increased levels of IL1β-induced *IL6* and *IL8* mRNA expression.

## Results

### RNA-Seq Transcriptome Profiling of GC-treated Primary Human ASM Cells

To identify GC-responsive genes in ASM, we performed RNA-Seq expression profiling of primary ASM cells from four white male donors treated with 1 µM dexamethasone (DEX) or control vehicle for 18 h, a treatment protocol that captures a large set of genes regulated by the GR [17]. We obtained an average of 58.9 million raw sequencing reads per sample (range 44.2–71.3 million reads per sample). Of these reads, an average of 83.36% were aligned to hg19 genome reference files downloaded from Illumina's iGenomes project (range 81.94%–84.34%) [Table S1]. An average of 26.43% of the mapped reads spanned junctions. Most bases in mapped reads corresponded to mRNA (>98%) [Table S2]. Plots of normalized read coverage of transcripts vs. normalized position, reveals that there was even coverage of transcripts by reads [Figure S1]. Based on these and various quality control (QC) summary metrics, including ERCC spike-in dose response plots, the sequencing and alignment results for each sample were deemed of sufficiently high quality to include in differential expression analyses. Quantification of transcript and gene expression levels was performed using Cufflinks according to hg19 RefSeq annotation files from Illumina's iGenomes Project.

**Figure 1:**
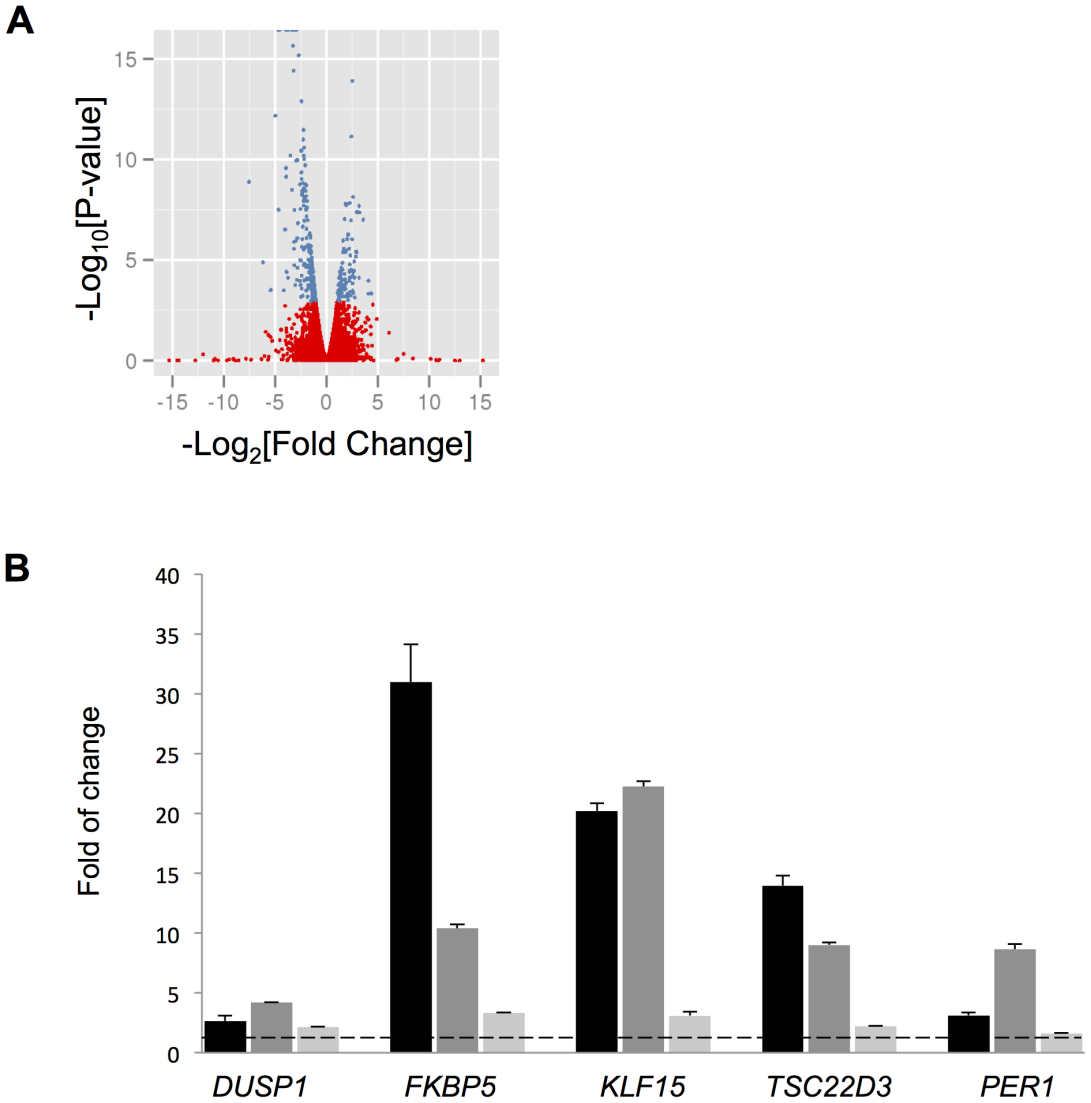
RNA-Seq profiling of DEX-treated ASM cells. **A**) Volcano plot of overall gene-based differential expression results for four cell lines treated with DEX vs. left untreated (each dot corresponds to a gene). The y-axis corresponds to the negative log (base 10) of P-values while the x-axis corresponds to the negative log (base 2) of the fold change for difference in expression when cells were stimulated with DEX. There were 316 differentially expressed genes according to an adjusted p-value <0.05 (blue dots). **B**) Validation of known GC-responsive genes through qRT-PCR. After ASM cells were treated with 1 µM DEX for 18 h, the mRNA levels of indicated genes were measured by qRT-PCR and the folds of change in mRNA induced by DEX were calculated. Each column bar represents an individual cell line. Experiments for each cell line were performed in triplicate, and the error bars are SE values corresponding to a cell line's replicates. The dotted line indicates a fold change of 1. ** *P*<0.005, * *P*<0.05 (t test).

Overall, 316 genes were significantly differentially expressed after correcting for false discovery rate by the Benjamini-Hochberg [23] approach [Figure 1A, Table S3]. Table 1 contains the genes with Q-value <1E-10 that were considered for further study. Some of these top genes have been previously related to steroid responsiveness and inflammation (i.e., *DUSP1* [*24*], *FKBP5* [25], *KLF15* [*17*], *PER1* [*12*], [*26*], and *TSC22D3* [*25*], [27]), and their upregulation by 1 µM for 18 DEX was confirmed by quantitative real time PCR (qRT-PCR) in ASM cells from three donors [Figure 1B]. qRT-PCR results for the fourth donor used in the RNA-Seq experiment were also consistent [Figure S2]. Other genes identified via the RNA-Seq experiment were considered potentially novel GC-responsive genes as they have little published evidence regarding a relationship with steroid responsiveness and/or inflammation. Gene set enrichment analysis using the NIH DAVID tool [28] identified various Gene Ontology and other annotation categories that were overrepresented by the 316 genes. The top six gene functional annotation clusters (enrichment scores >3) had terms related to: glycoprotein/extracellular matrix, vasculature development, circulatory system process, response to nutrients, thrombospondin type-1, and response to hormone stimulus terms [Table S4]. Other clusters among the 19 with enrichment scores >1.5 that may be relevant to lung disease included lung development, regulation of cell migration, and extracellular matrix organization.

**Table 1:** Top (Q-value <1E-10) differentially expressed genes.

Gene	locus	Mean FPKM Control	Mean FPKM Dex	Ln[ Fold Change ]	P-value	Q-value
*C7*	chr5:40909598-40983042	3.76	38.41	3.35	<1E-16	<1E-16
*CCDC69*	chr5:150560612-150603654	6.24	47.39	2.92	<1E-16	<1E-16
*DUSP1*	chr5:172195092-172198203	18.26	144.96	2.99	<1E-16	<1E-16
*FKBP5*	chr6:35541361-35704724	3.43	53.05	3.95	<1E-16	<1E-16
*GPX3*	chr5:150399998-150408554	45.18	613.37	3.76	<1E-16	<1E-16
*KLF15*	chr3:126061477-126076236	0.86	20.46	4.58	<1E-16	<1E-16
*MAOA*	chrX:43515408-43606068	4.41	43.24	3.29	<1E-16	<1E-16
*SAMHD1*	chr20:35504569-35580246	17.27	245.78	3.83	<1E-16	<1E-16
*SERPINA3*	chr14:95078713-95090390	13.78	139.89	3.34	<1E-16	<1E-16
*SPARCL1*	chr4:88394487-88450655	1.07	27.88	4.70	<1E-16	<1E-16
*C13orf15*	chr13:42031541-42045013	10.00	96.35	3.27	2.2E-16	2.5E-13
*TSC22D3*	chrX:106956451-107019017	9.69	93.26	3.27	2.2E-16	2.5E-13
*CRISPLD2*	chr16:84853586-84943116	7.89	51.17	2.70	6.7E-16	6.9E-13
*PER1*	chr17:8043787-8055753	1.49	13.69	3.20	3.8E-15	3.6E-12
*KCTD12*	chr13:77454303-77460540	31.25	5.43	-2.52	1.2E-14	1.1E-11
*ERRFI1*	chr1:8071778-8086393	13.48	72.70	2.43	1.3E-13	1.1E-10
*STEAP4*	chr7:87905743-87936228	0.20	6.35	4.99	6.7E-13	5.3E-10

FPKM  =  fragments per kilobase of transcript per million mapped reads.

### Verification of GC-responsive Genes by q-PCR

**Figure 2:**
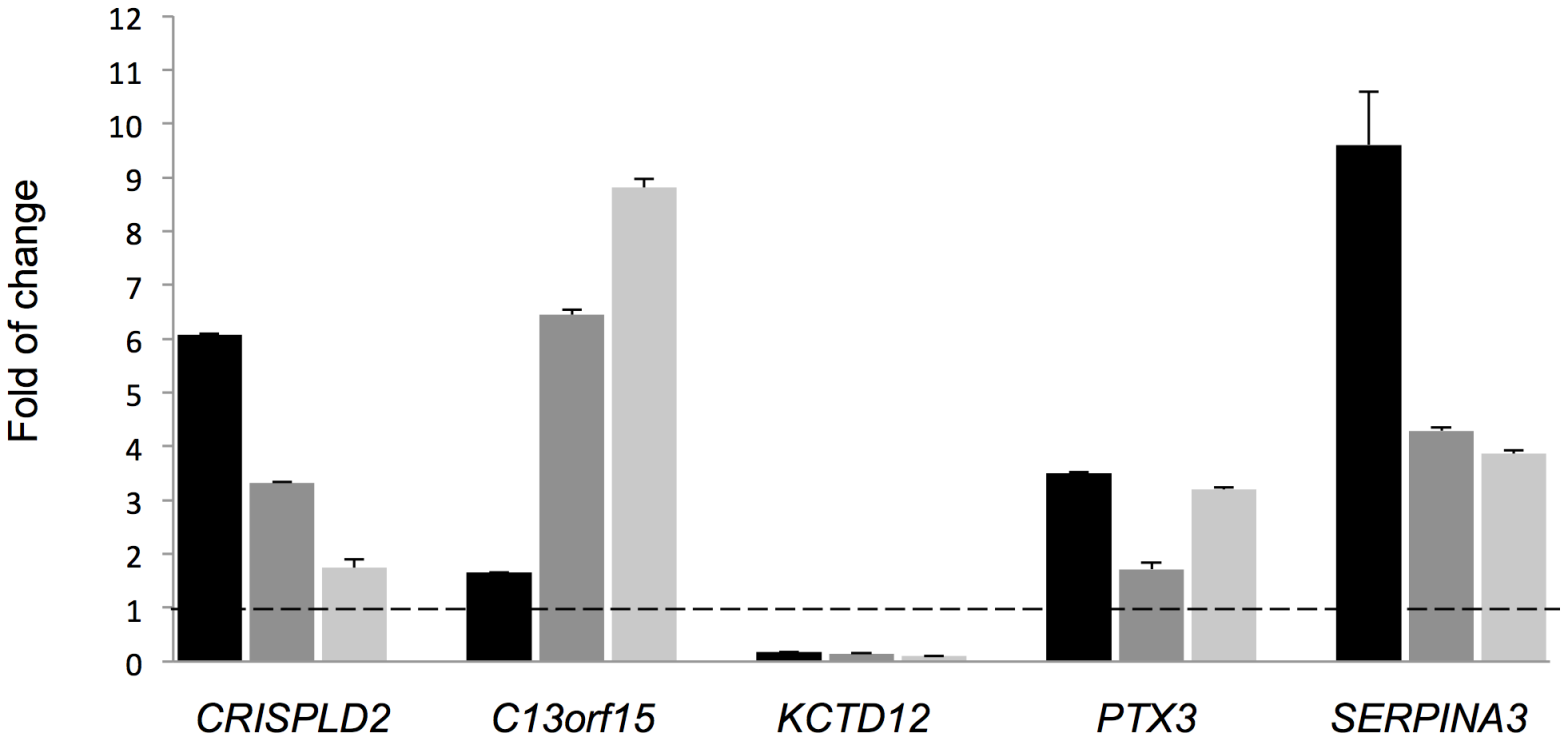
Validation of GC responsive genes. After cells from three individual ASM lines were treated with 1 µM DEX for 18 h, the mRNA levels of indicated genes were measured by qRT-PCR and the folds of change induced by DEX were calculated. Each column bar represents an individual cell line; experiments for each cell line were performed in triplicate. Error bars are SE values corresponding to a cell line's replicates.

A subset of the top differentially expressed genes (i.e., *CRISPLD2, C13orf15, KCTD12, SERPINA3*) was selected for follow-up based on each gene's potential to be a novel steroid responsiveness gene. Differential expression for these four genes and one additional gene selected from the top 316 differentially expressed ones (i.e., *PTX3*) was verified via qRT-PCR by treating with 1 µM DEX for 18 h three of the ASM cell lines used for RNA-Seq [Figure 2] to compare biological sample variability and effect sizes obtained via RNA-Seq vs. qRT-PCR. qRT-PCR results for the fourth donor were consistent with those for the other three cell lines [Figure S2]. Gene expression levels varied among the primary cell lines, suggesting an inherent heterogeneity in individual GC responsiveness. Nevertheless, the qRT-PCR data for each of the genes was consistent in direction of fold-change with the RNA-Seq results.

### *CRISPLD2* Variants Associated with Asthma Pharmacogenetic Phenotypes

Inhaled corticosteroid (ICS) responsiveness is a measure of improvement in pulmonary function after treatment with a glucocorticoid. To determine whether any of the differentially expressed genes were associated with this pharmacogenetic phenotype, defined as unchanged improvement in lung function among asthma patients after receiving ICS therapy for 4–8 weeks, we obtained previously conducted ICS GWAS results (unpublished) for SNPs within, or spanning 50 kb on either side, each of the genes in Table 1. Based on a threshold of 1E-03, the *CRISPLD2* gene had SNPs that were nominally associated with ICS resistance [Table 2; Figure S3]. Because the beta-agonist and glucocorticoid pathways are known to overlap [29], we also examined the association of the differentially expressed genes with bronchodilator response, which measures the effect of beta-agonists on lung function. Based on bronchodilator response GWAS results from a previous study where the phenotype was defined as change in FEV_1_ in response to administration of the beta-agonist albuterol [30], SNPs in *CRISPLD2* and an additional gene *CCDC69* were nominally associated with the bronchodilator response [Table 2; Figure S3]. Additionally, replication results for one SNP (rs8047416) from this bronchodilator response GWAS that had a primary P-value of 4.5E-04 had been obtained for 552 white subjects from the Severe Asthma Research Program (SARP) cohort and found to have a P-value of 0.038 (overall P-value 9.0E-05). Together these results suggest a role for *CRISPLD2* in modulating two asthma pharmacogenetic phenotypes.

**Table 2:** SNPs within 50Table 1 that are associated (overall P-value <1E-03) with bronchodilator response (BDR) or inhaled corticosteroid (ICS) resistance in human clinical trial cohorts.

CHR	SNP	BP	A1	A2	A1 FREQ	P-value	Gene	Phenotype
5	rs13155012	150546065	G	A	0.93, 0.94, 0.94	7.6E-04	*CCDC69*	BDR
16	rs58151657	84867507	G	A	0.85, 0.88, 0.88	1.6E-04	*CRISPLD2*	BDR
16	rs8047416	84871409	C	T	0.88, 0.88, 0.88	4.4E-04	*CRISPLD2*	BDR
16	rs7189551	84958011	T	C	0.76, 0.76, 0.72, 0.78	7.8E-04	*CRISPLD2*	ICS Resistance
16	rs7188498	84958018	G	A	0.76, 0.75, 0.71, 0.78	5.1E-04	*CRISPLD2*	ICS Resistance
16	rs9928433	84958414	A	G	0.78, 0.80, 0.78, 0.84	5.8E-04	*CRISPLD2*	ICS Resistance
16	rs67343076	84964590	G	A	0.76, 0.78, 0.77, 0.83	3.3E-04	*CRISPLD2*	ICS Resistance
16	rs8061778	84986714	G	T	0.86, 0.84, 0.84, 0.79	7.5E-04	*CRISPLD2*	ICS Resistance

A1 FREQ lists frequencies from individual cohorts used to compute overall P-value for each phenotype.

### *CRISPLD2* Expression Changes in Previous Microarray Studies of the ASM GC Response

We analyzed publicly available data from two published gene expression microarray studies (GSE34313 [17] and GSE13168 [18]) that measured the effect of GCs on human ASM cells to determine whether these previous studies supported our *CRISPLD2* differential expression results. Although *CRISPLD2* did not rank as one of the most highly differentially expressed genes in these studies, all comparisons available between ASM cells treated with a GC vs. baseline conditions demonstrate that *CRISPLD2* had significant adjusted P-values [Table 3]. Specifically, the GSE34313 study found that *CRISPLD2* was differentially expressed both 4 and 24 hours after ASM cells were treated with DEX, and the GSE13168 study found that the differential *CRISPLD2* expression was strongest when ASM cells were treated with a GC (i.e. fluticasone) vs. left untreated, than when cells were also stimulated with pro-inflammatory cytokines (i.e. EGF and IL1β).

**Table 3:** Differential expression results for *CRISPLD2* obtained from publicly available data of two previous microarray studies that investigated the effects of GCs on human ASM cells.

Study	Comparison Group	Rank	Adjusted P-value	Fold Change
GSE34313	4 hr DEX vs. baseline	305	5.1E-05	1.85
GSE34313	24 hr DEX vs. baseline	850	8.3E-06	1.95
GSE13168	Fluticasone vs. basal	6	3.9E-07	6.09
GSE13168	Fluticasone+EGF vs. EGF	4	2.4E-05	4.33
GSE13168	Fluticasone+IL1β vs. IL1β	45	1.5E-03	2.78
GSE13168	Fluticasone+EGF+IL1β vs. EGF+IL1β	68	3.9E-02	2.13

Rank refers to ranking within all differentially expressed genes of each comparison group.

### GC Induced *CRISPLD2* mRNA and Protein Expression in Primary Human ASM Cells

**Figure 3:**
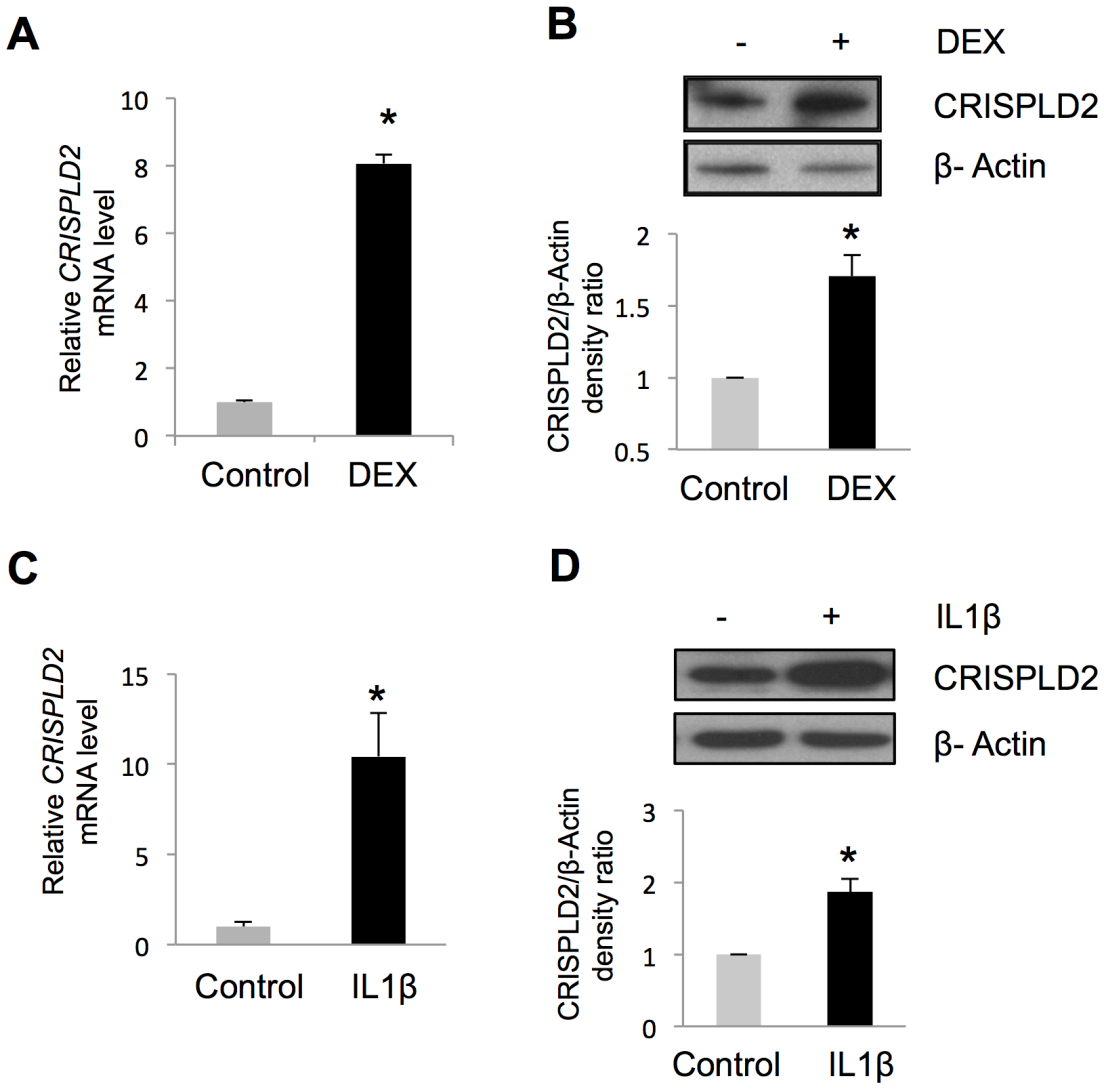
*CRISPLD2* is a GC- and IL1β-responsive gene. ASM cells were treated with 100**A**) increased *CRISPLD2* mRNA expression as measured by qRT-PCR, **B**) increased *CRISPLD2* protein expression as measured by immuno-blotting. ASM cells were treated with 5 ng/mL IL1β for 24 h, resulting in **C**) increased *CRISPLD2* mRNA expression as measured by qRT-PCR, and **D**) increased *CRISPLD2* protein expression as measured by immuno-blotting. *CRISPLD2* mRNA levels were measured in triplicate. CRISPLD2 protein levels are shown as normalized blot densitometry values, and the error bars are SE values across three independent experiments. * *P*<0.05 (*t* test).

Because of its potential to modulate two important asthma drug response phenotypes vis-à-vis these associations and published evidence of its involvement in lung development and endotoxin regulation [31], we focused our functional studies on the *CRISPLD2* gene to investigate its potential role in steroid and immune response in ASM cells. We grew the most GC sensitive ASM cell line among those tested in Figure 2, treated those cells with DEX, and extracted RNA for qRT-PCR and protein for immune-blot analysis. Upon DEX treatment, *CRISPLD2* mRNA increased 8.1-fold [Figure 3A]. Consistent with mRNA changes, protein levels of *CRISPLD2* in ASM cells also increased upon DEX treatment by 1.7-fold at 24 hours [Figure 3B]. Using cells from a single donor, the effect of DEX on *CRISPLD2* expression was found to be time [Figure S4A] and dose dependent [Figure S4B]. The induction of *CRISPLD2* by DEX that was observed in ASM did not occur in A549 pulmonary epithelial cells derived from a lung carcinoma tissue, as analogous treatment of A549 cells with DEX caused a decrease of *CRISPLD2* mRNA [Figure S5].

### *CRISPLD2* is Induced by IL1β and Modulates the Expression of Two Immuno-Responsive Genes

**Figure 4:**
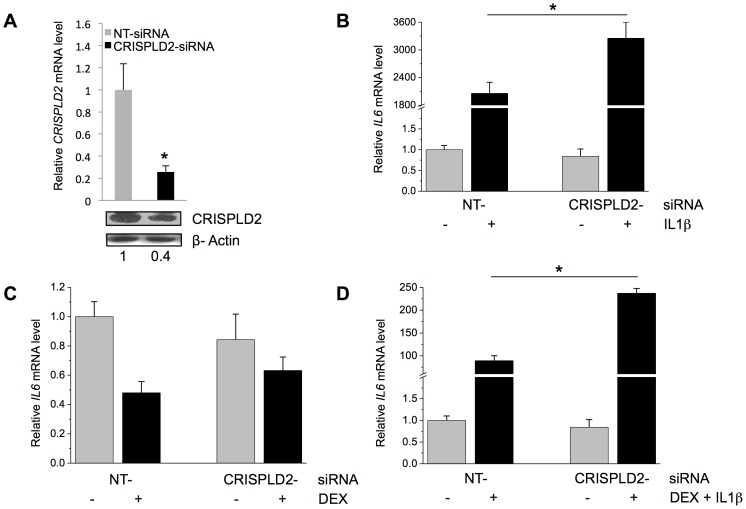
*CRISPLD2* regulates the response to inflammatory cytokines. **A**) Effect of *CRISPLD2*-specific siRNA on *CRISPLD2* mRNA and protein levels. ASM cells were transfected with *CRISPLD2*-specific siRNA or non-targeting (NT) siRNA, and 72 h later, *CRISPLD2* mRNA and protein levels were determined by qRT-PCR (levels normalized to those in control cells transfected with NT siRNA) and immuno-blotting, respectively. The effect of *CRISPLD2* knockdown on IL1β-induced cytokine expression was assessed by transfecting ASM cells with *CRISPLD2*-specific or NT siRNA, and 72 h later, treating cells for 24 h with **B**) 5 ng/mL IL1β, **C**) 100 nM DEX, or **D**) 5 ng/mL IL1β and 100 nM DEX. *IL6* expression was determined by qRT-PCR. Normalized mRNA levels are shown. Experiments were performed in triplicate, and the error bars are SE values for three samples. * *P*<0.05 (*t* test).

Because GCs exert anti-inflammatory effects, we tested the role of GC-induced *CRISPLD2* expression in regulating inflammatory responses in the ASM. Treatment of a single ASM cell line with the proinflammatory cytokine IL1β (5 ng/mL for 24 h) increased *CRISPLD2* mRNA by 10.4-fold and protein levels by 1.9-fold [Figure 3C and 3D], suggesting that *CRISPLD2* is not only GC-inducible but also immuno-responsive. We next performed knockdown experiments to assess whether *CRISPLD2* modulates IL1β-induced cytokine responses using a single ASM cell line. Because IL1β acts as an important mediator of inflammatory responses by activating other cytokines, we investigated the role of *CRISPLD2* in IL1β-induced expression of other known immune-response genes (i.e. *IL6* [32] and *IL8* [33]). In ASM cells transfected with *CRISPLD2*-specific siRNA, *CRISPLD2* mRNA expression was decreased by 74% and protein levels decreased by 60% [Figure 4A].

While expression levels of *IL6* did not change in response to *CRISPLD2* knockdown, treatment of ASM cells with IL1β induced significantly higher expression of *IL6* in *CRISPLD2*-knockdown cells as compared to NT siRNA control cells [Figure 4B], suggesting that *CRISPLD2* is an inhibitory modulator of immuno-response in ASM cells. Consistent with this notion, another cytokine's (i.e. *IL8*'s) induction by IL1β was also enhanced by *CRISPLD2* knockdown [Figure S6]. To further characterize the effect of *CRISPLD2* on immune response, we treated cells with 100 nM DEX alone or in combination with 5 ng/mL IL1β. *IL6* expression was decreased with DEX treatment, but *CRISPLD2* knockdown did not significantly change the *IL6* response to DEX [Figure 4C]. However, *IL6* mRNA levels in *CRISPLD2* knockdown cells were higher than that in NT siRNA control cells when IL1β and DEX were administered simultaneously [Figure 4D], again supporting a role for *CRISPLD2* in modulating IL1β response.

### *CRISPLD2* is Not Required for the Expression of GC Target Genes

We performed siRNA-mediated *CRISPLD2* knockdown experiments to assess whether *CRISPLD2* affects the transcriptional expression of well-known GC-response genes (i.e. *DUSP1* [*24*] *, FKBP5* [25] or *TSC22D3* [*25*], [*27*]). The expression levels of three GR target genes were not significantly altered in *CRISPLD2*-knockdown ASM cells relative to control non-targeting siRNA-transfected cells [Figure S7], suggesting that *CRIPSLD2* does not modulate the direct transcriptional actions of GCs in the ASM.

## Discussion

Due to their widespread use in the treatment of various inflammatory diseases and the notoriety of their undesirable side effects, GCs have been extensively characterized for their effects on specific tissues [3], [34]. In the case of asthma, an important target of GCs is the ASM [13]. Our RNA-Seq, qRT-PCR and immuno-blot results demonstrated that *CRISPLD2* is a GC responsive gene, with DEX increasing its mRNA and protein levels. We found that *CRISPLD2* mRNA and protein levels increase in response to treatment with a known pro-inflammatory cytokine (IL1β), and that *CRISPLD2* knockdown increased the IL1β responsiveness of two inflammatory genes (i.e. *IL6* and *IL8*), suggesting that *CRISPLD2* may regulate immune response. Specifically, *CRISPLD2* may interfere with IL1β-induced cytokine production and act to reduce immune response via a negative feedback loop that can be activated by IL1β. This negative feedback loop may also play a role in cytokine level modulation in response to DEX treatment, as evidenced by the increased levels of *IL6* observed when both IL1β and DEX were administered to *CRISPLD2* knockdown ASM cells vs. when DEX was administered alone.

The *CRISPLD2* gene maps to chromosome 16 at 16q24.1, covering 118.32 kb. According to AceView [35], *CRISPLD2* is highly expressed in many human tissues, including lung and trachea. While AceView describes 14 different mRNAs as transcript products for this gene, only one of these (NM_031476) was part of the RefSeq annotation file used in the RNA-Seq analysis. Based on mapping of raw reads, each exon of this reported mRNA isoform was expressed in our ASM samples [Figure S8]. Results obtained after repeating the alignment and transcript reconstruction with parameters that allow for the discovery of novel isoforms while using the reference hg19 genome as a guide, also suggested that a single *CRISPLD2* mRNA isoform (NM_031476) was present in the ASM samples. Further studies are required to find out whether *CRISPLD2* splicing variation occurs in different individuals, at rare frequencies, and/or under other biological conditions.

The cell-specific effects of GCs are well known [5] and understanding the mechanisms of such specificity is an active area of research [12], [36]. For example, a study of the cell-specific responsiveness of *PTX3* to GCs found that in fibroblasts and endothelial cells, the GR functioned as a ligand-dependent transcription factor to induce *PTX3* gene expression, while in macrophages and myeloid dendritic cells, the GR repressed *PTX3* transcription by interfering with the action of other signaling pathways [37]. Comparison of baseline *CRISPLD2* expression in ASM vs. A549 pulmonary epithelial cells revealed that *CRISPLD2* is more highly expressed in ASM [Figure S5A]. This finding is consistent with the results from Reddy *et al*, who found low levels of *CRISPLD2* mRNA in A549 cells [12]. While ASM *CRISPLD2* levels increased in response to DEX, levels decreased in A549 cells according to our experiments [Figure S5B] and were not significantly changed after 100 nM DEX treatment for 1 hour in the Reddy *et al* [12] study. In a study using BEAS-2B cells, *CRISPLD2* expression was induced by both a glucocorticoid (fluticasone) and a long-acting beta-agonist (formoterol) [38]. Further studies are required to understand the cell-specific expression of *CRISPLD2* and its interactions with other asthma medications.

A hypothesis of the mechanism by which GCs activate *CRISPLD2* in ASM is provided by the chromatin immunoprecipitation followed by DNA sequencing (ChIP-Seq) results from Reddy *et al*, who sought to identify regions of the genome where GRs bind at various concentrations of DEX (100 nM 50 nM, 5 nM, 500 pM) [12]. Their results, which are part of the Transcription Factor ChIP-Seq V4 results from ENCODE [39], in/near *CRISPLD2* indicate that a region between its transcription start site (TSS) and first exon binds GRs [Figure S8]. While Reddy *et al* did not further characterize the GR binding of *CRISPLD2* because this gene was not DEX-responsive in their study, our RNA-Seq results along with their ChIP-Seq results suggest that GCs may activate *CRISPLD2* in ASM by binding to GR enhancer regions. However, future ChIP experiments are necessary to validate the exact location and sequence of this potential GR-binding site that may increase *CRISPLD2* expression in ASM cells.

CCAAT/Enhancer Binding Proteins (CEBPs) are a family of transcription factors that help regulate a wide variety of processes, including inflammatory response [40]. Two of these factors, *CEBPB* and *CEBPD*, have been shown to be induced by GCs in lung epithelium [41] and skeletal muscle cells [42]. Additionally, the transcription of *CEBPB* has been shown to be induced by IL1, IL6, and lipopolysaccharide (LPS) [40]. ENCODE Transcription Factor ChIP-Seq V4 results suggest that CEBPs may also induce transcription of *CRISPLD2* [Figure S8]. Specifically, two CEBPB binding regions detected in untreated A549, HepG2 (hepatocellular carcinoma), IMR90 (fetal lung fibroblasts), and K562 (immortalized chronic myelogenous leukemia) cells between the TSS and first exon of *CRISPLD2* are salient as they have cluster scores of 1000 out of 1000 (all others near/in *CRISPLD2* have scores of <435). According to our RNA-Seq data, both *CEBPB* and *CEBPD* were expressed in ASM [Figure S9], but only *CEBPD* had significantly increased mRNA levels in response to treatment with DEX [Q-value 4.8E-04, Ln of fold-change 1.47]. Thus, if DEX does indeed mediate changes in expression of *CRISPLD2* in the ASM via CEBPB binding, it likely does so via a post-translational mechanism such as by changing phosphorylation [43]. Future ChIP experiments are necessary to confirm that CEBPB binding contributes to *CRISPLD2* expression in ASM.

The potential presence of both GR and CEBPB binding sites in *CRISPLD2* is consistent with our observation that both DEX and IL1β increased *CRISPLD2* mRNA expression, findings that may seem at odds because one mechanism by which GCs act is to decrease expression of cytokines such as IL1β. Because IL1 induces *CEBPB* expression [44], IL1β may induce transcription of *CRISPLD2* via increased binding of CEBPB to a CEBPB transcription factor in *CRISPLD2* in the absence of a GC [Figure 3C]. The control ASM cell lines used to obtain RNA-Seq results expressed low levels of *IL1β* both at baseline (FPKM  = 0.04) and when treated with DEX (FPKM  = 0.01), and thus, we did not characterize the relationships among DEX, *IL1β* and *CRISPLD2* that would be expected of a disease involving high levels of IL1β. Further studies examining changes of expression of *CRISPLD2* under varying concentrations of DEX and IL1βwould help to clarify the relationships among them.

*CRISPLD2* (a.k.a. *Lgl1* in rat) has been identified as a developmental gene that modulates branching morphogenesis in fetal rat lung [45] and its variants have been related to non-syndromic cleft lip with or without cleft palate in human association studies [46]. In a recent study of CRISPLD2's role in endotoxin regulation, CRISPLD2 was found to bind to LPS, thereby preventing LPS from binding target peripheral blood mononuclear cells (PBMCs) and inhibiting the release of proinflammatory markers (i.e. TNFα and IL6) by these target cells [31]. Endogenous CRISPLD2 in healthy human serum was found to downregulate LPS-induced TNFα production *in vitro*, and CRISPLD2 was found to protect mice from endotoxic shock. A subsequent study of CRISPLD2 found that its protein levels were decreased in blood serum of patients with septic shock compared to controls, patients with sepsis and patients with severe sepsis [47]. However, CRISPLD2 levels were not related to clinical outcomes (e.g., survival). Although their use is highly controversial, GCs have been used to treat septic shock [48], and thus, one area of future study could be to characterize the relationships among GCs, CRISPLD2 and LPS in septic shock. Our findings that *CRISPLD2* knockdown increased *IL6* and *IL8* levels, while DEX increased *CRISPLD2* levels, taken together with the study by Wang, *et al* demonstrating that CRISPLD2 plays a role in endotoxin regulation, suggest that CRISPLD2, in part, may modulate asthma phenotypes by decreasing the ASM inflammatory response to exogenous LPS-containing bacteria. However, the role of LPS and endotoxin in the development of asthma or its exacerbations is not fully understood [49], and further studies are needed to test potential roles of *CRIPSLD2* in linking GCs and inflammation to endotoxin regulation.

In addition to published evidence that *CRISPLD2* may indirectly play a role in asthma, SNPs of this gene were associated with two asthma pharmacogenetic traits measured in asthma clinical trials. Each of these traits was relevant to GC response and ASM contractility: ICS resistance was a direct measure of treatment with a glucocorticoid, while bronchodilator response was a measure of beta-agonist effects, and the beta-agonist and glucocorticoid pathways are known to overlap [29]. Interestingly, the region of association with BDR overlaps with the GR and CEBPB DNA-binding regions mentioned above [Figure S10]. In a previous study, the long-acting beta-agonist formoterol was found to activate a CEBP-luciferase reporter construct in BEAS-2B cells, and mice with a lung epithelial-specific knockout of CEBPB were found to have an impaired suppression of LPS-induced neutrophilia by formoterol compared to control littermates [50]. Thus, it is possible that this region of association with bronchodilator response reflects a functional change that alters GR and/or CEBPB binding, but further experiments are required to test this hypothesis. While the nominal associations with ICS resistance and bronchodilator response do not reach genome-wide significance, and hence, would not suggest that *CRISPLD2* variants be prioritized for further study based on the GWAS data alone, in the context of the current GC responsiveness results, they suggest that specific regions in/near *CRISPLD2* may modulate asthma phenotypes in humans.

Results from two publicly available gene expression microarray studies that have measured the effect of GCs on human ASM cells using *in vitro* models supported our *CRISPLD2* findings [Table 3]. The first study by Masuno, *et al* (GSE34313) investigated the effects of DEX at 4 and 24 hours and focused on the functional validation of the *KLF15* gene, which was found to modulate airway hyperresponsiveness, but not inflammatory response, in an ovalbumin challenge mouse asthma model [17]. While not among their top-ranked findings, *CRISPLD2* expression was increased by DEX at both 4 and 24 hours in the experiments by Masuno, *et al.* Consistent with our findings, DEX increased the expression of *CRISPLD2* at both 4 and 24 hours in the experiments by Masuno, *et al.* Consistent with the results of Masuno, *et al*, *KLF15* was among the top differentially expressed genes that we identified. Another microarray study of the ASM transcriptome by Misior *et al* (GSE13168) focused on the overlap of GC and beta-agonist gene responses [18]. Of most relevance to our work, *CRISPLD2* had significantly increased expression levels when ASM cells were treated with fluticasone. Further, the effect of fluticasone on *CRISPLD2* expression was diminished when ASM were also treated with IL1β and/or EGF pro-inflammatory cytokines [Table 3]. This suggests that *CRISPLD2* levels were increased by both EGF and IL1β and is consistent with our result that IL1β increased *CRISPLD2* expression [Figures 3C and 3D].

While *in vitro* studies of the ASM response to GCs that use RNA-Seq have not been published, a recent study used RNA-Seq to investigate the effects of a 2-week course of oral prednisolone on ASM gene expression in patients with mild asthma, using ASM extracted via laser caption microdissection from bronchoscopy samples [51]. Comparing samples from 6 patients assigned to GC treatment vs. 6 patients assigned to placebo, this study found that 15 genes were significantly differentially expressed between groups, and two of the 15 genes were also associated with airway hyperresponsiveness. Of these 15 genes, only one was significant in our study (i.e. *SYNPO2*, adjusted P-value 0.015) [Table S3]. Future studies of the *in vivo* GC response of ASM may help clarify the differences between *in vitro* and *in vivo* study results.

Our study has identified well-known and more novel GC responsive genes, but it was also subject to limitations. We used cell lines from four white male subjects, and based on both RNA-Seq and qRT-PCR results, there was significant variability in gene expression levels among subjects. For example, the qRT-PCR data in Figure 2 suggests that there is an inverse correlation between levels of *CRISPLD2* and *C13orf15*. That is, DEX seemed to induce greater levels *CRISPLD2* in cells that had lower levels of *C13orf15*. Because the cell lines used were derived from “an outbred human population,” the heterogeneity of responses is consistent with the complexity of the pathophysiology of asthma. Future studies with a larger number of individuals and individuals of diverse gender and racial/ethnic backgrounds may shed light on individualized profiles of GC response, including identifying individuals who are GC insensitive, and increase our understanding of how the expression of various genes relates to each other. Our RNA-Seq analyses were limited to the hg19 RefSeq annotation files downloaded from Illumina's iGenomes project. Thus, we did not characterize the expression long-non-coding RNA or mRNA transcript isoforms that were not part of the reference file used. We opted for use of a well-annotated reference file for our investigation of the ASM transcriptome to reduce the number of false-positive results that we may have selected for follow-up. Future studies with more comprehensive annotation files and a greater number of individuals and/or greater sequencing depth will yield additional insight into the ASM transcriptome.

While ASM is a target tissue in the GC treatment of asthma, our ASM samples were not from asthma patients. Although the study by Masuno *et al* found that there was general concordance between response to DEX in 16 genes among four control ASM cell lines and those of two asthma patients [17], there are likely some differences in the GC response between asthma patients and individuals without asthma. Further studies that include ASM from asthma patients may help clarify such differences. Finally, it is known that the response to GCs changes in time. For example, the Masuno *et al* study compared the ASM GC response at both 4 and 24 hours, and it found that while some genes had consistent changes at both time points, others had varied ones [17]. We selected an 18-hour DEX treatment period partly because the study by Masuno *et al* suggested that the set of genes regulated by the GR expands greatly between 4 and 24 hours. Future studies that evaluate the GC response over time would further define how GCs alter the ASM transcriptome.

In summary, we identified 316 GC responsive genes in primary ASM cell lines. The *CRISPLD2* gene was selected for functional studies based on having SNPs that were nominally associated with ICS resistance and bronchodilator response, as well as having published studies relating it to lung development and endotoxin response. Based on results of *in vitro* experiments, *CRISPLD2* was found to be a modulator of IL1β response in ASM cells. Our findings identified *CRISPLD2* as a novel asthma pharmacogenetics candidate gene and provide transcriptome data to further explore the anti-inflammatory effects of GCs in the ASM.

## Materials and Methods

### Ethics Statement

Lung tissue was obtained from the National Disease Resource Interchange (NDRI) and its use approved by the University of Pennsylvania Institutional Review Board; use of the cells does not constitute human research. Approval of the GWAS studies was issued by the Partners Healthcare, Inc. Partners Human Research Committee, which ensured that all procedures followed were in accordance with the ethical standards of the responsible committee on human experimentation, including obtaining written informed consent for all study participants.

### ASM Cell Culture and GC Treatment for RNA-Seq Experiment

Primary ASM cells were isolated from four white aborted lung transplant donors with no chronic illness. ASM cell cultivation and characterization were described previously [52], [53]. Passages 4 to 7 ASM cells maintained in Ham's F12 medium supplemented with 10% FBS were used in all experiments. For the RNA-Seq and qRT-PCR validation experiments, cells from each donor were treated with 1 µM DEX (Sigma-Aldrich, St. Louis, MO) or control vehicle for 18 h. For other experiments, cells were treated with 100 nM DEX for 24 or 48 h.

### RNA-Seq Library Construction and Sequencing

Total RNA was extracted from control and DEX-treated ASM cells using the miRNAeasy mini kit (Qiagen Sciences, Inc., Germantown, MD). Approximately 1 µg of RNA from each sample was used to generate RNA-Seq cDNA libraries for sequencing using the TruSeq RNA Sample Prep Kit v2 (Illumina, Inc., San Diego, CA). Sample preparation followed the manufacturer's protocol with a workflow that included isolation of poly-adenylated RNA molecules using poly-T oligo-attached magnetic beads, enzymatic RNA fragmentation, cDNA synthesis, ligation of bar-coded adapters, and PCR amplification. Ambion External RNA Controls Consortium (ERCC) RNA Spike-In Control Mix 1 (Life Technologies Corporation, Carlsbad, CA) was added to the samples. The amplified cDNA fragments were analyzed using the 2100 Bioanalyzer (Agilent Technologies, Inc., Santa Clara, CA) to determine fragment quality and size. Library concentrations were determined by Qubit Fluorometric Quantitiation (Life Technologies Corporation, Carlsbad, CA). Sequencing of 75 bp paired-end reads was performed with an Illumina HiSeq 2000 instrument at the Partners HealthCare Center for Personalized Genetic Medicine (Boston, MA).

### RNA-Seq Data Analysis

Preliminary processing of raw reads was performed using Casava 1.8 (Illumina, Inc., San Diego, CA). Subsequently, Taffeta scripts (https://github.com/blancahimes/taffeta) were used to analyze RNA-Seq data, which included use of FastQC [54] (v.0.10.0) to obtain overall QC metrics. Based on having sequence bias in the initial 12 bases on the 5′ end of reads, the first 12 bases of all reads were trimmed with the FASTX Toolkit (v.0.0.13) [55]. FastQC reports for each sample revealed that each was successfully sequenced. Trimmed reads for each sample were aligned to the reference hg19 genome and known ERCC transcripts using TopHat [56] (v.2.0.4), while constraining mapped reads to be within reference hg19 or ERCC transcripts. Additional QC parameters were obtained to assess whether reads were appropriately mapped. Bamtools [57] was used to the number of mapped reads, including junction spanning reads. The Picard Tools (http://picard.sourceforge.net) RnaSeqMetrics function was used to compute the number of bases assigned to various classes of RNA, according to the hg19 refFlat file available as a UCSC Genome Table. For each sample, Cufflinks [21] (v.2.0.2) was used to quantify ERCC Spike-In and hg19 transcripts based on reads that mapped to the provided hg19 and ERCC reference files. For three samples that contained ERCC Spike-Ins, we created dose response curves (i.e. plots of ERCC transcript FPKM vs. ERCC transcript molecules) following the manufacturer's protocol [58]. Ideally, the slope and R^2^ would equal 1.0. For our samples (Dex.2, Control.4, Dex.4), the slope (R^2^) values were 0.90 (0.90), 0.92 (0.84), 0.82 (0.86), respectively. Raw read plots were created by displaying bigwig files for each sample in the UCSC Genome Browser.

Differential expression of genes and transcripts in samples treated with DEX vs. untreated samples was obtained using Cuffdiff [21] (v.2.0.2) with the quantified transcripts computed by Cufflinks (v.2.0.2), while applying bias correction. The CummeRbund [59] R package (v.0.1.3) was used to measure significance of differentially expressed genes and create plots of the results. As a positive control of gene expression, the FPKM values for four housekeeping genes (i.e., *B2M*, *GABARAP*, *GAPDH*, *RPL19*) were obtained. Each had high FPKM values that did not differ significantly by treatment status [Figure S11]. The NIH Database for Annotation, Visualization and Integrated Discovery (DAVID) was used to perform gene functional annotation clustering using Homo Sapiens as background, and default options and annotation categories (Disease: OMIM_DISEASE; Functional Categories: COG_ONTOLOGY, SP_PIR_KEYWORDS, UP_SEQ_FEATURE; Gene_Ontology: GOTERM_BP_FAT, GOTERM_CC_FAT, GOTERM_MF_FAT; Pathway: BBID, BIOCARTA, KEGG_PATHWAY; Protein_Domains: INTERPRO, PIR_SUPERFAMILY, SMART) [28]. The RNA-Seq data is available at the Gene Expression Omnibus Web site (http://www.ncbi.nlm.nih.gov/geo/) under accession GSE52778.

### Genome-wide Association Studies

Two GWAS of asthma-related traits that may be related to ASM GC response were selected to measure association of SNPs in/near top differentially expressed genes identified by RNA-Seq:

1. A GWAS of inhaled corticosteroid (ICS) response was conducted in 723 non-Hispanic white asthmatics from the following drug clinical trials: Childhood Asthma Management Program (CAMP) [60], Leukotriene Modifier or Corticosteroid Salmeterol study (LOCCS) [61], and subsets of trials within the Childhood Asthma Research and Education (CARE) network [62], and the Asthma Clinical Research Network (ACRN) [63] participating in the NHLBI SNP Health Association Resource (SHARe) Asthma Resource project (SHARP). ICS response was quantified as the percent change in pre-BD FEV_1_ following 4–8 weeks of ICS therapy [i.e. (onICSpreFEV_1_ – offICSpreFEV_1_)/offICSpreFEV_1_)]. Association of imputed SNPs (based on the June 2010 release of the 1000 Genome Project reference) with ICS response was measured using a linear regression model using PLINK [64].
2. Previously, a GWAS of bronchodilator response was conducted in 1,644 non-Hispanic white asthmatics from drug clinical trials [30]. Briefly, bronchodilator response was quantified as the percent change in FEV_1_ in response to administration of a β_2_-agonist bronchodilator (BD) [i.e. (post-BD FEV_1_ – pre-BD FEV_1_)/pre-BD FEV_1_], and association of single nucleotide polymorphisms (SNPs) with bronchodilator response was measured using a linear regression model, while adjusting for age, sex, and height. Results for 4,571,615 imputed SNPs (based on the June 2010 release of the 1000 Genome Project reference) were available.

Originally computed hg18 coordinates were converted to those of the hg19 human genome assembly using the liftOver tool of the UCSC genome browser. Variant names were converted to those of the dbSNP build 138.

### Publicly Available GC-treated ASM Microarray Expression Data Analysis

Datasets from two publicly available gene expression microarray studies that measured the effect of GCs on human ASM cells using *in vitro* models were obtained from the Gene Expression Omnibus (GEO): GSE34313 and GSE13168. Raw signals of the GSE34313 experiment, which used four replicates of a single ASM cell line to investigate the effects of DEX at 4 and 24 hours using the Agilent-014850 Whole Human Genome Microarray 4x44K platform [17], were preprocessed with background correction and quantile normalization using the limma R package [65]. The GSE13168 dataset consisted of ASM cell cultures extracted from four donor tracheas that were stimulated with a GC (fluticasone) or a protein kinase A inhibitor as well as pro-inflammatory agents (i.e. EGF and/or IL1β) or control and used the Affymetrix U133A platform to measure gene expression changes [18]. Its raw signal intensities were preprocessed with RMA as implemented in the affy R package [66]. For both datasets, gene-based differential expression analysis was conducted using the limma R package by averaging probe intensities for individual genes [65].

### Cell culture, Chemical Treatment and siRNA Transfection

ASM cells were maintained in Ham's F12 medium supplemented with 24 mM HEPES, 1.7 mM CaCl2, 12 Mm NaOH and 10% FBS. For chemical treatment, cells grown in the above medium were washed with PBS and switched to the serum deprivation medium. A549 human lung epithelial cells were maintained in high-glucose DMEM medium containing 10% FBS. For chemical treatment, A549 cells were washed and switched to DMEM/F12(1∶1) medium supplemented with 3% dialyzed FBS. 100 nM DEX (Sigma-Aldrich Corporation, St. Louis, MO) or 5 ng/mL IL1β (Invitrogen, Life Technologies, Grand Island, NY) was then added to the medium. Transfection of *CRISPLD2* and non-targeting siRNA (siRNA universal non-targeting control 1, Sigma-Aldrich Corporation, St. Louis, MO) was performed using DharmaFECT 1 reagent according to the recommended protocol from the manufacturer (Thermo Scientific, Lafayette, CO). The final concentration of siRNA was 25 nM; siRNA sequences for *CRSIPLD2* knockdown were 5′-GAACCAACAUCUAUGCAGA(dT)(dT)-3′ and 5′-UCUGCAUAGAUCUUGGUUC(dT)(dT)-3′.

### Quantitative Real-Time PCR (qRT-PCR) Analysis

Total RNA was isolated from cells by using QIAshredder and RNeasy kits (Qiagen Sciences, Inc., Germantown, MD). Oligo(dT)-primed cDNA was prepared from 50 ng of total RNA by using SuperScript III First-strand Synthesis System (Invitrogen, Life Technologies, Grand Island, NY). qRT-PCR was set up in the presence of 0.5 µM primers by using QuantiTect SYBR Green PCR kit (Qiagen Sciences, Inc., Germantown, MD). qRT-PCR was performed on an StepOne Plus real time PCR machine (Applied Biosystems, Life Technologies, Grand Island, NY). β-actin was used as an internal control for data normalization. Each sample was measured in triplicate.

### Immunoblot Analysis

Cells were washed with PBS and lysed in NP-40 lysing buffer (50 mM Tris-HCl pH 7.5, 150 mM NaCl_2_, 0.5% Nonidet P-40) containing protease inhibitor cocktail (Roche, Genentech, Inc., South San Francisco, CA). For the secreted *CRISPLD2* protein analysis, the medium was concentrated using Ultra-15 10 K Centrifugal Filter Devices (Merck Millipore, Tullagreen, IRL). Protein samples were separated on NuPAGE 4–12% Bis-Tris gels (Invitrogen, Life Technologies, Grand Island, NY) and transferred to PVDF membranes (Bio-Rad, Life Science Research, Hercules, CA). Immunoblot signals were developed using SuperSignal West Pico Chemiluminescent Substrates (Pierce Biotechnology, Inc., Rockford, IL). Primary antibodies used in the study included rabbit polyclonal CRISPLD2 antibody (Abcam, Cambridge, MA) and mouse monoclonal β-actin antibody (Santa Cruz Biotechnology, Inc., Dallas, TX). Relative sample intensities were computed with scanned and quantified immunoblot data obtained using Image J software [67]. Each Western blot experiment was performed 3 times; representative image results are shown. Semi-quantitative results are reported as the mean +/− SE.

## Supporting Information

Figure S1 **Estimated read coverage across transcripts for each sample.** Position and coverage are normalized by adjusting for transcript lengths and total number of reads mapped per sample.
(PNG)

Figure S2 **Confirmation of qRT-PCR results supporting RNA-Seq findings in the ASM cell line that was not used for validation in** **Figure 1B** **or** **Figure 2** **.** The mRNA levels of the indicated genes were measured by qRT-PCR and the folds of change induced by DEX were calculated for a single replicate.
(TIFF)

Figure S3 **Association of SNPs near** ***CRISPLD2*** **with A) bronchodilator response and B) ICS resistance.** The x-axes denote position along Chromosome 16 according to the hg18 genome build. The y-axes denote –Log_10_(P) corresponding to 1000GP imputed data P-values. LD between the SNP with the lowest P-values (chr16:83425008 and chr16:83522091) to each SNP in the plot is denoted in colors and was computed according to 1000GP June 2010 CEU data. Plot was created using LocusZoom [68].
(TIFF)

Figure S4 **Time and dose dependent effects of DEX on CRISPLD2 expression.** **A**) ASM cells were treated with 100 nM DEX for 24 and 48 h. **B**) ASM cells were treated with DEX at indicated concentrations for 24 h. CRISPLD2 protein was measured by immune-blotting.
(TIFF)

Figure S5 **DEX induced expression change of** ***CRISPLD2*** **in A549 pulmonary epithelial cells.** **A**) Basal mRNA level of *CRISPLD2* in A549 and ASM cells. **B**) A549 cells were treated with 100 nM DEX for 24 h and *CRISPLD2* mRNA levels were measured by qRT-PCR. Relative values of gene expression shown. Experiment was performed in triplicate using cells from a single donor. ** *P*<0.005, * *P*<0.05 (*t* test).
(TIFF)

Figure S6 ***CRISPLD2*** **regulates** ***IL8*** **expression.** ASM cells were transfected with *CRISPLD2*-specific siRNA or non-targeting (NT) siRNA, and 72 h later cell were treated with 5 ng/mL IL1β for 24 h. *IL8* mRNA expression was determined by qRT-PCR. Normalized mRNA levels shown. All measurements were performed in triplicate samples. * *P*<0.05 (*t* test).
(TIFF)

Figure S7 **Effect of** ***CRISPLD2*** **knockdown on GR target gene expression.** ASM cells were first transfected with *CRISPLD2*-specific or NT siRNA and then stimulated with 100 nM DEX for 24 h. Induced expression (DEX treatment vs. control) of three GR target genes was determined by qRT-PCR. None of the target genes were found to be differentially expressed (i.e. all had t-test *P*>0.05).
(TIFF)

Figure S8 **Raw RNA-Seq read plots for four ASM cell lines treated with DEX (red) or left untreated (blue) along the human (hg19) chromosome 16 region containing** ***CRISPLD2*** **.** Reads mapped to each exon of the RefSeq version of CRISPLD2. An increased number of mapped reads is observed in each sample after DEX treatment vs. when left untreated. Shown below the RefSeq gene track are ENCODE ChIP-Seq tracks: four are for sites found to bind the GR (official gene name *NR3C1*) in A549 pulmonary epithelial cells at various dosages of DEX (100 nM 50 nM, 5 nM, 500 pM) [12], and below these are Transcription Factor ChIP-Seq V4 results for GR, CEBPB, and CEBPD. Darker vertical lines represent binding sites with higher scores (i.e. detected more strongly).
(TIFF)

Figure S9 **RNA-Seq results from four ASM cell lines treated with DEX expressed as FPKM for** ***CEBPB*** **and** ***CEBPD*** **by condition status (i.e. DEX vs. untreated) show the presence of both genes.** DEX treatment did not significantly change the expression levels of *CEBPB* but did change the expression levels of *CEBPD* (Q-value 4.8E-04, Ln of fold-change 1.47).
(TIFF)

Figure S10 **Region of** ***CRISPLD2*** **where SNPs were most strongly associated with bronchodilator response (BDR) along with GR- and CEBPB-binding sites identified by ENCODE Transcription Factor ChIP-Seq V4 results.** The x-axis denotes position along Chromosome 16 in hg19 genome build coordinates. The vertical axis of the BDR GWAS Results denotes –Log_10_(P-values), and the horizontal line at 1.3 represents a nominal significance threshold of P-value = 0.05. Shown below the RefSeq gene track are ENCODE ChIP-Seq tracks for sites found to bind the GR (official gene name *NR3C1*) in A549 pulmonary epithelial cells at various dosages of DEX (100 nM 50 nM, 5 nM, 500 pM) [12], and below these are Transcription Factor ChIP-Seq V4 results for GR, CEBPB, and CEBPD. Darker ChIP-Seq regions represent binding sites with higher scores (i.e. detected more strongly).
(TIFF)

Figure S11 **RNA-Seq results from four ASM cell lines treated with DEX expressed as FPKM for four housekeeping genes (i.e.** ***B2M*** **,** ***GABARAP*** **,** ***GAPDH*** **,** ***RPL19*** **) by condition status (i.e. DEX vs. untreated) show high levels of expression for each gene that did not significantly differ with DEX treatment.**
(TIFF)

Table S1 Number of various read types per sample in millions. Values in parenthesis for unmapped and mapped reads correspond to percentages of total reads per sample, while values in parenthesis for all other entries correspond to percentages of total mapped reads per sample.
(DOCX)

Table S2 Percentage of mapped bases according to hg19 reference refFlat file base type.
(DOCX)

Table S3 Significantly differentially expressed genes.
(DOCX)

Table S4 Functional annotation clusters obtained with the NIH DAVID tool using differentially expressed genes in Table S3. Clusters with enrichment scores >1.5 are shown. Individual P-values listed correspond to EASE Scores, or modified Fisher Exact P-Values computed by DAVID.
(DOCX)
