# Supplementary figures and images for: RNA-Seq Transcriptome Profiling Identifies CRISPLD2 as a Glucocorticoid Responsive Gene that Modulates Cytokine Function in Airway Smooth Muscle Cells
Source: PLoS One. 2014 Jun 13;9(6):e99625. doi: 10.1371/journal.pone.0099625 (PMC4057123; doi:10.1371/journal.pone.0099625)

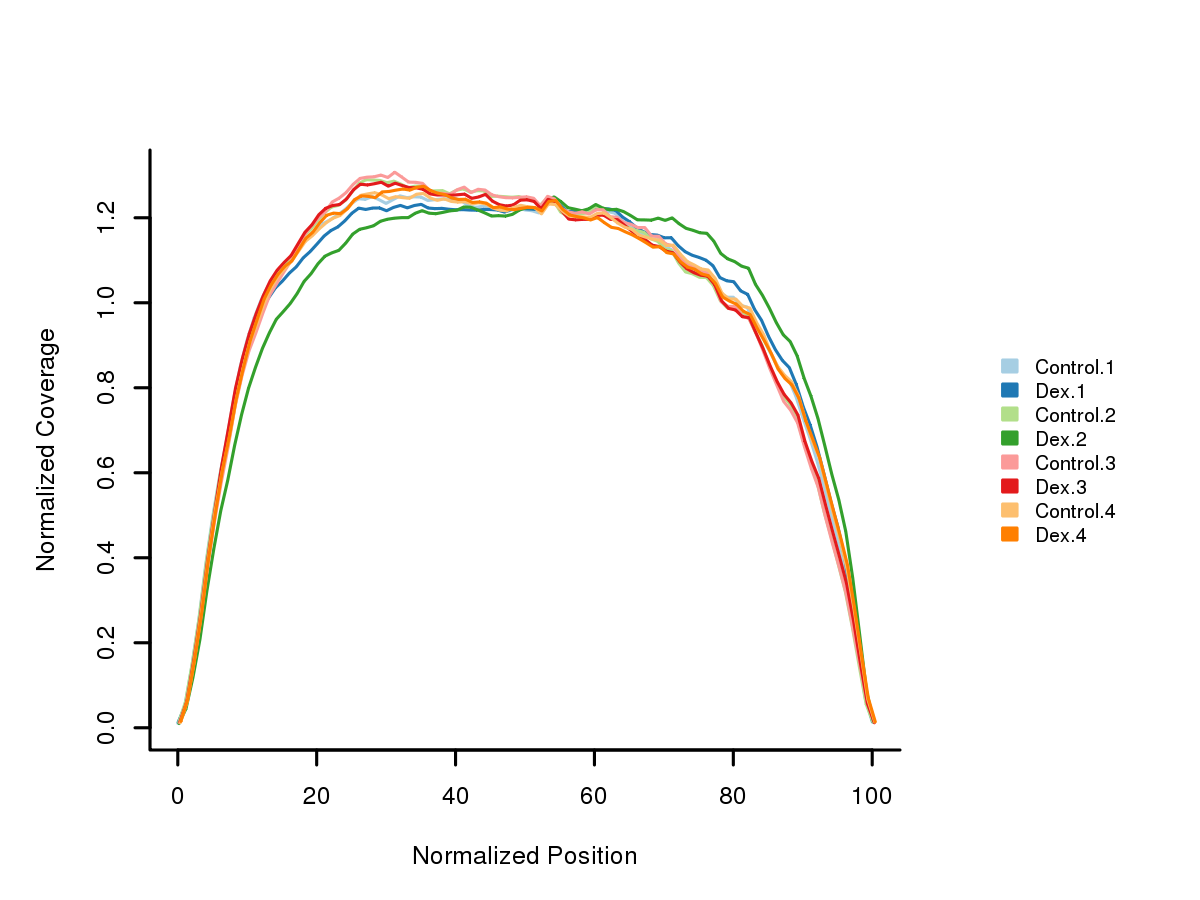

Supplement: Figure S1 — Estimated read coverage across transcripts for each sample. Position and coverage are normalized by adjusting for transcript lengths and total number of reads mapped per sample. (PNG) [file pone.0099625.s001.png]

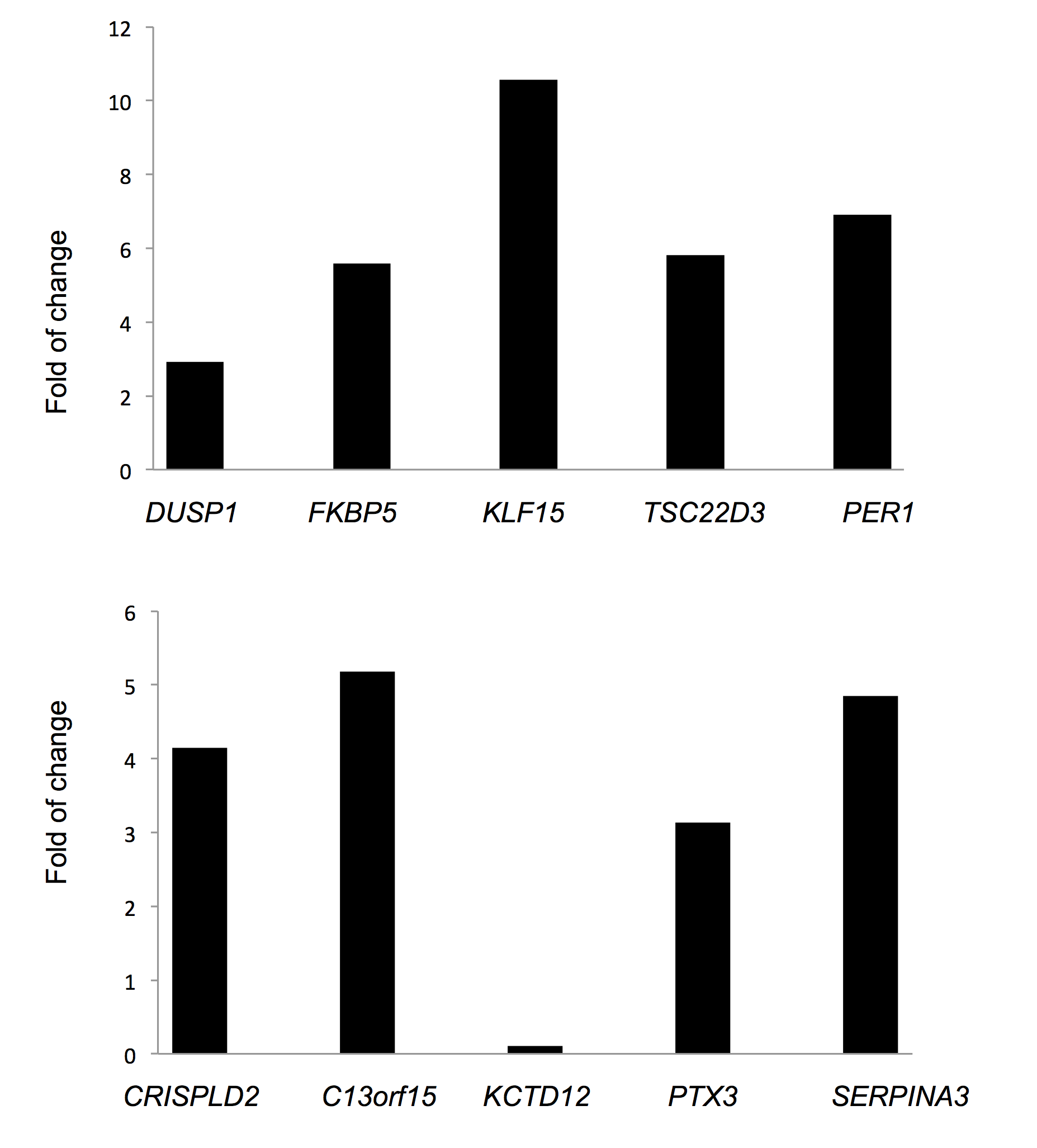

Supplement: Figure S2 — Confirmation of qRT-PCR results supporting RNA-Seq findings in the ASM cell line that was not used for validation in Figure 1B or Figure 2 . The mRNA levels of the indicated genes were measured by qRT-PCR and the folds of change induced by DEX were calculated for a single replicate. (TIFF) [file pone.0099625.s002.tif]

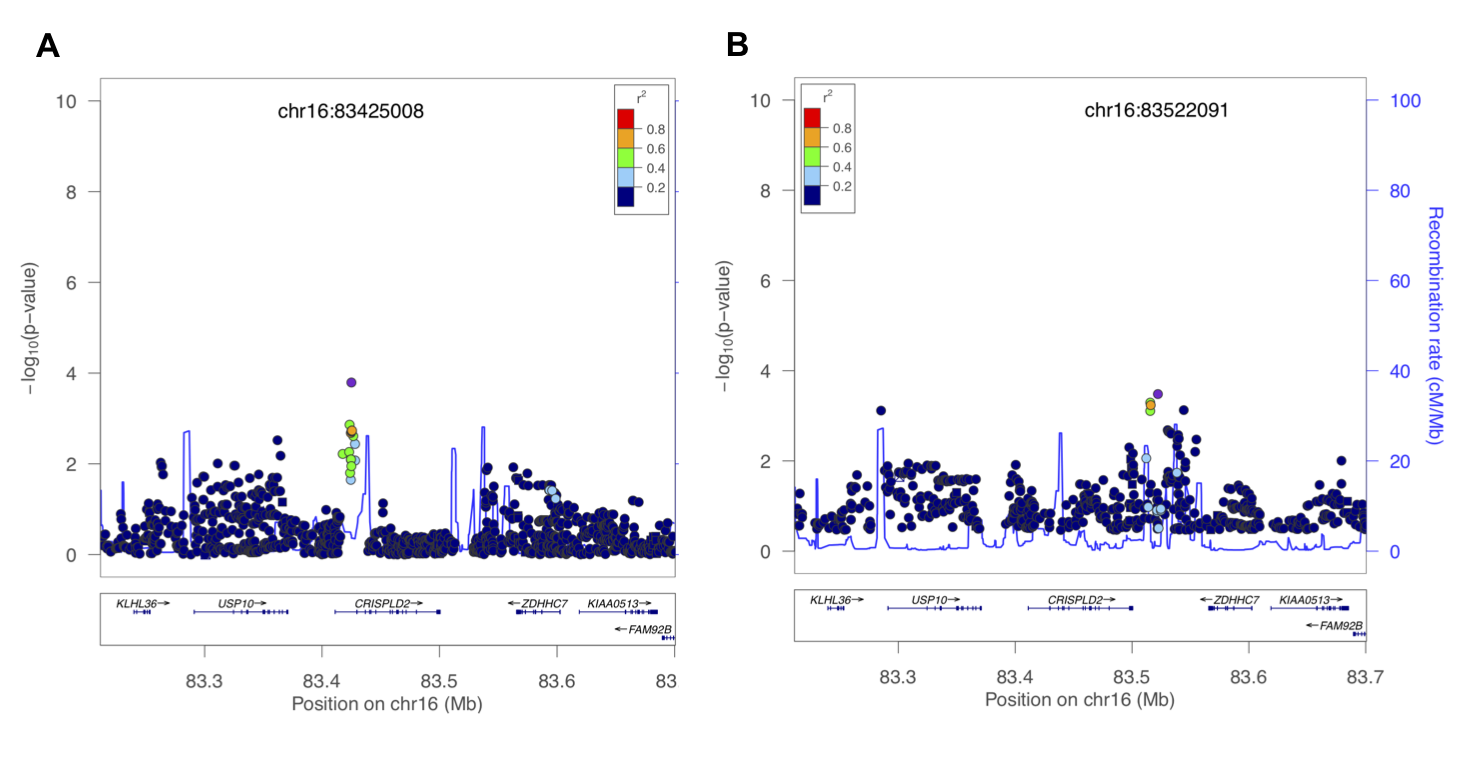

Supplement: Figure S3 — Association of SNPs near CRISPLD2 with A) bronchodilator response and B) ICS resistance. The x-axes denote position along Chromosome 16 according to the hg18 genome build. The y-axes denote –Log10(P) corresponding to 1000GP imputed data P-values. LD between the SNP with the lowest P-values (chr16:83425008 and chr16:83522091) to each SNP in the plot is denoted in colors and was computed according to 1000GP June 2010 CEU data. Plot was created using LocusZoom [68]. (TIFF) [file pone.0099625.s003.tif]

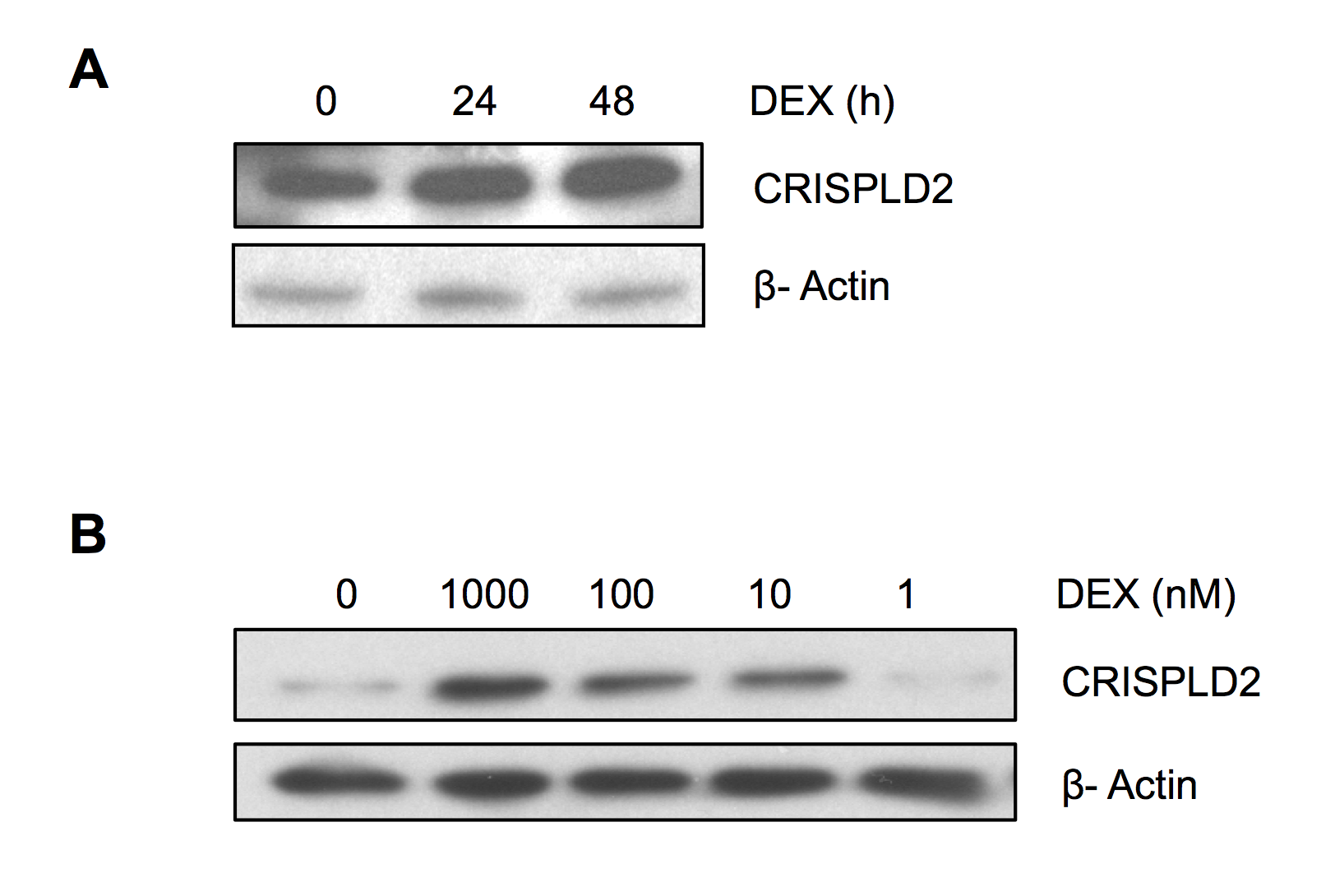

Supplement: Figure S4 — Time and dose dependent effects of DEX on CRISPLD2 expression. A) ASM cells were treated with 100 nM DEX for 24 and 48 h. B) ASM cells were treated with DEX at indicated concentrations for 24 h. CRISPLD2 protein was measured by immune-blotting. (TIFF) [file pone.0099625.s004.tif]

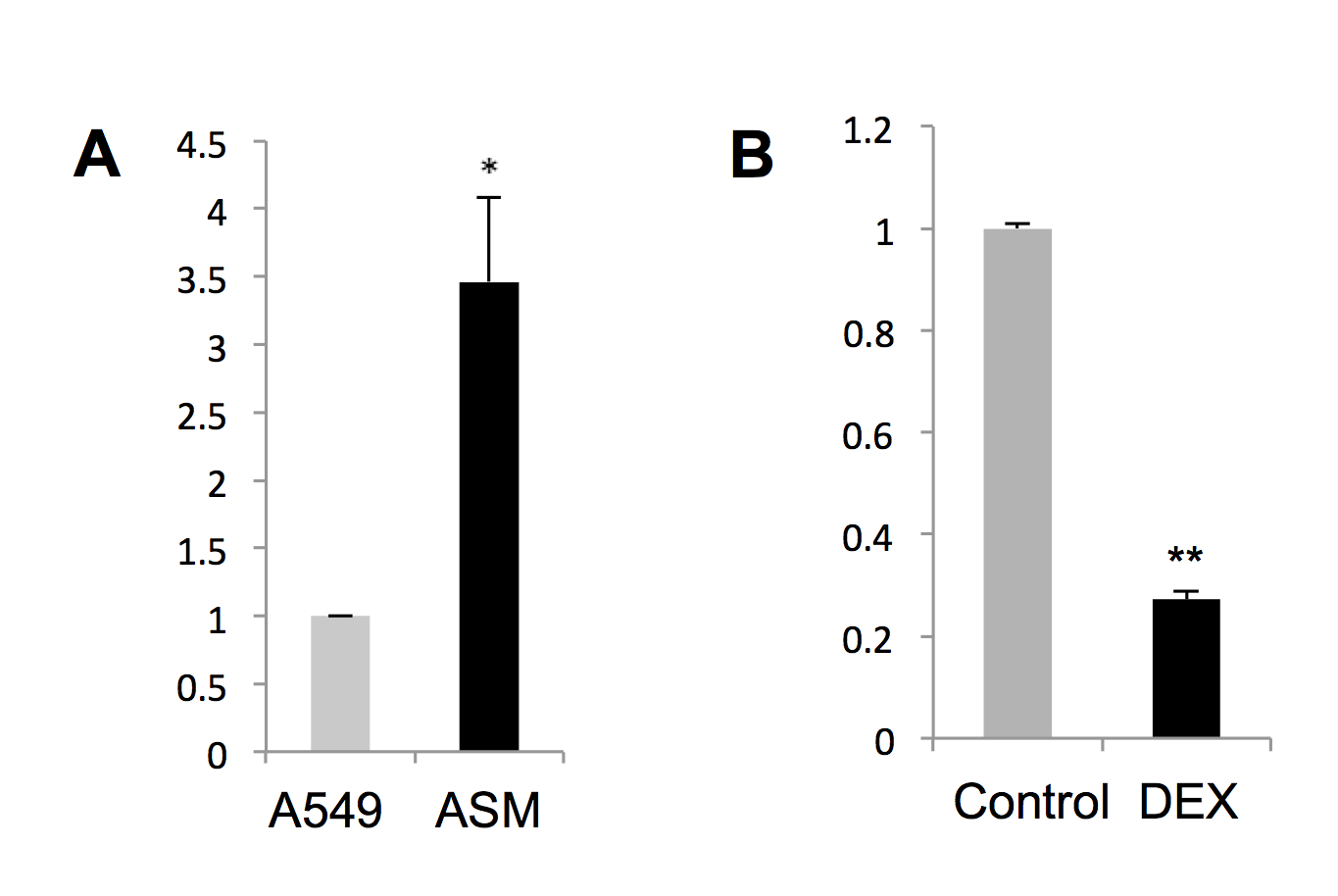

Supplement: Figure S5 — DEX induced expression change of CRISPLD2 in A549 pulmonary epithelial cells. A) Basal mRNA level of CRISPLD2 in A549 and ASM cells. B) A549 cells were treated with 100 nM DEX for 24 h and CRISPLD2 mRNA levels were measured by qRT-PCR. Relative values of gene expression shown. Experiment was performed in triplicate using cells from a single donor. ** P<0.005, * P<0.05 (t test). (TIFF) [file pone.0099625.s005.tif]

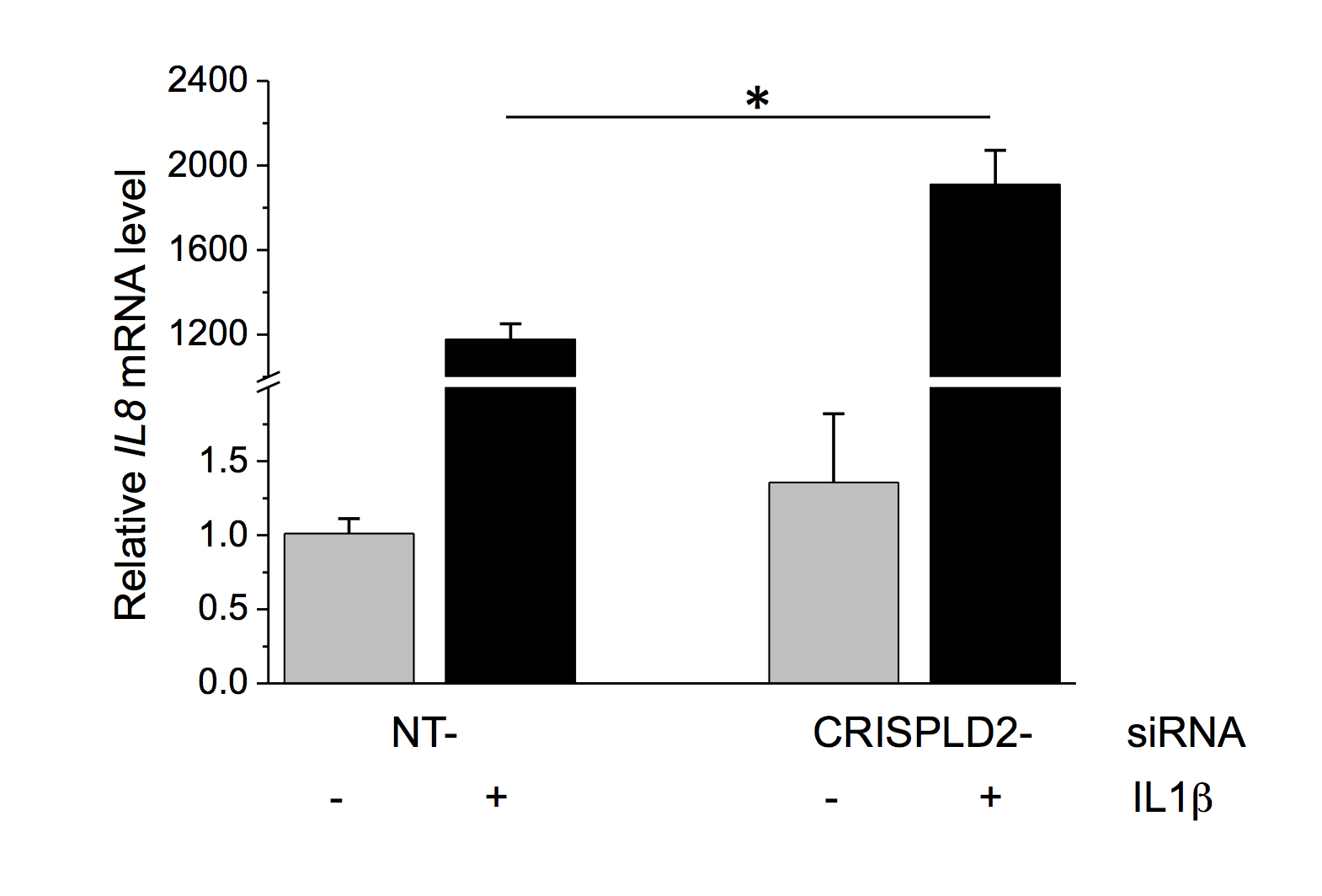

Supplement: Figure S6 — CRISPLD2 regulates IL8 expression. ASM cells were transfected with CRISPLD2-specific siRNA or non-targeting (NT) siRNA, and 72 h later cell were treated with 5 ng/mL IL1β for 24 h. IL8 mRNA expression was determined by qRT-PCR. Normalized mRNA levels shown. All measurements were performed in triplicate samples. * P<0.05 (t test). (TIFF) [file pone.0099625.s006.tif]

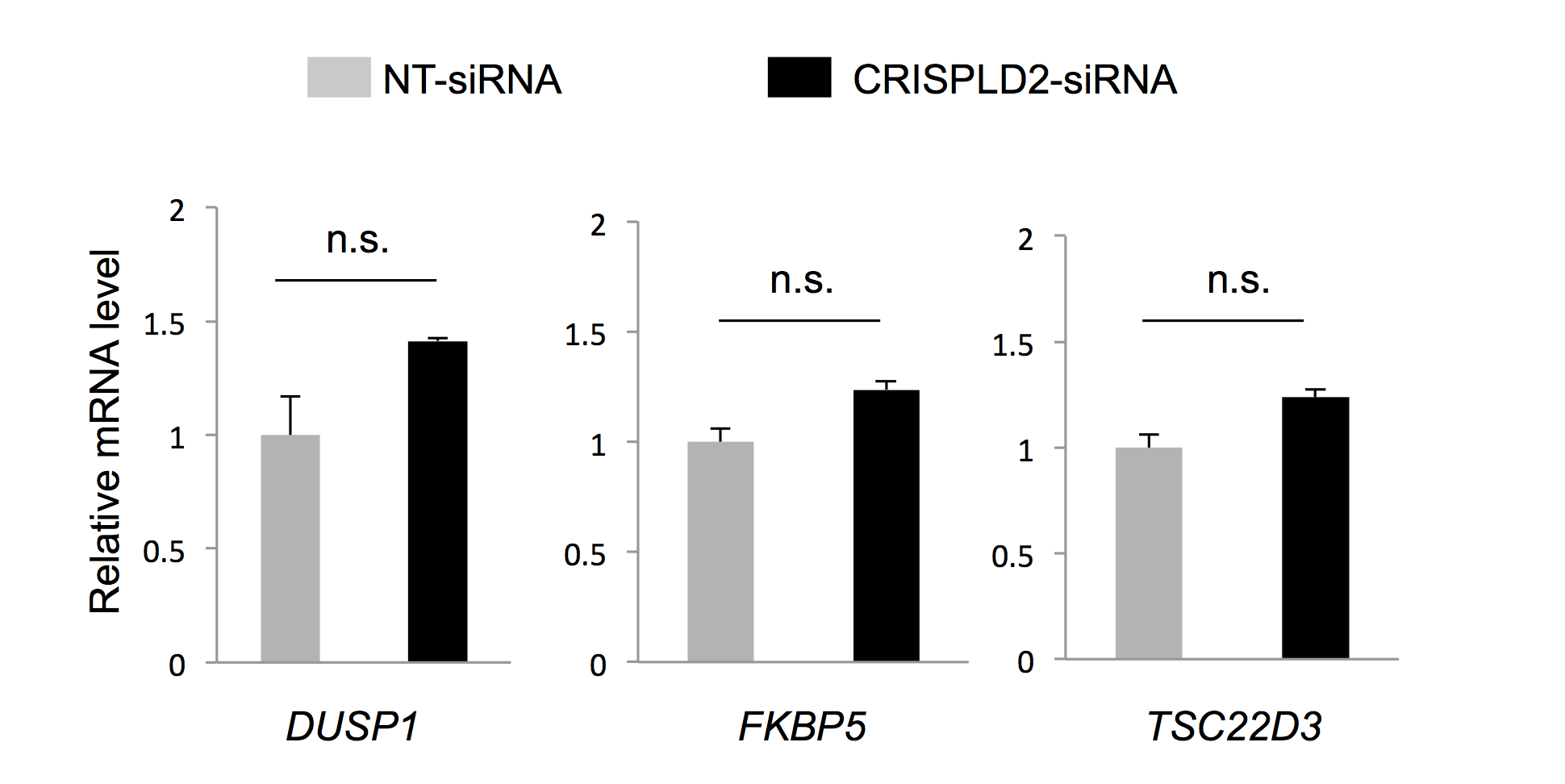

Supplement: Figure S7 — Effect of CRISPLD2 knockdown on GR target gene expression. ASM cells were first transfected with CRISPLD2-specific or NT siRNA and then stimulated with 100 nM DEX for 24 h. Induced expression (DEX treatment vs. control) of three GR target genes was determined by qRT-PCR. None of the target genes were found to be differentially expressed (i.e. all had t-test P>0.05). (TIFF) [file pone.0099625.s007.tif]

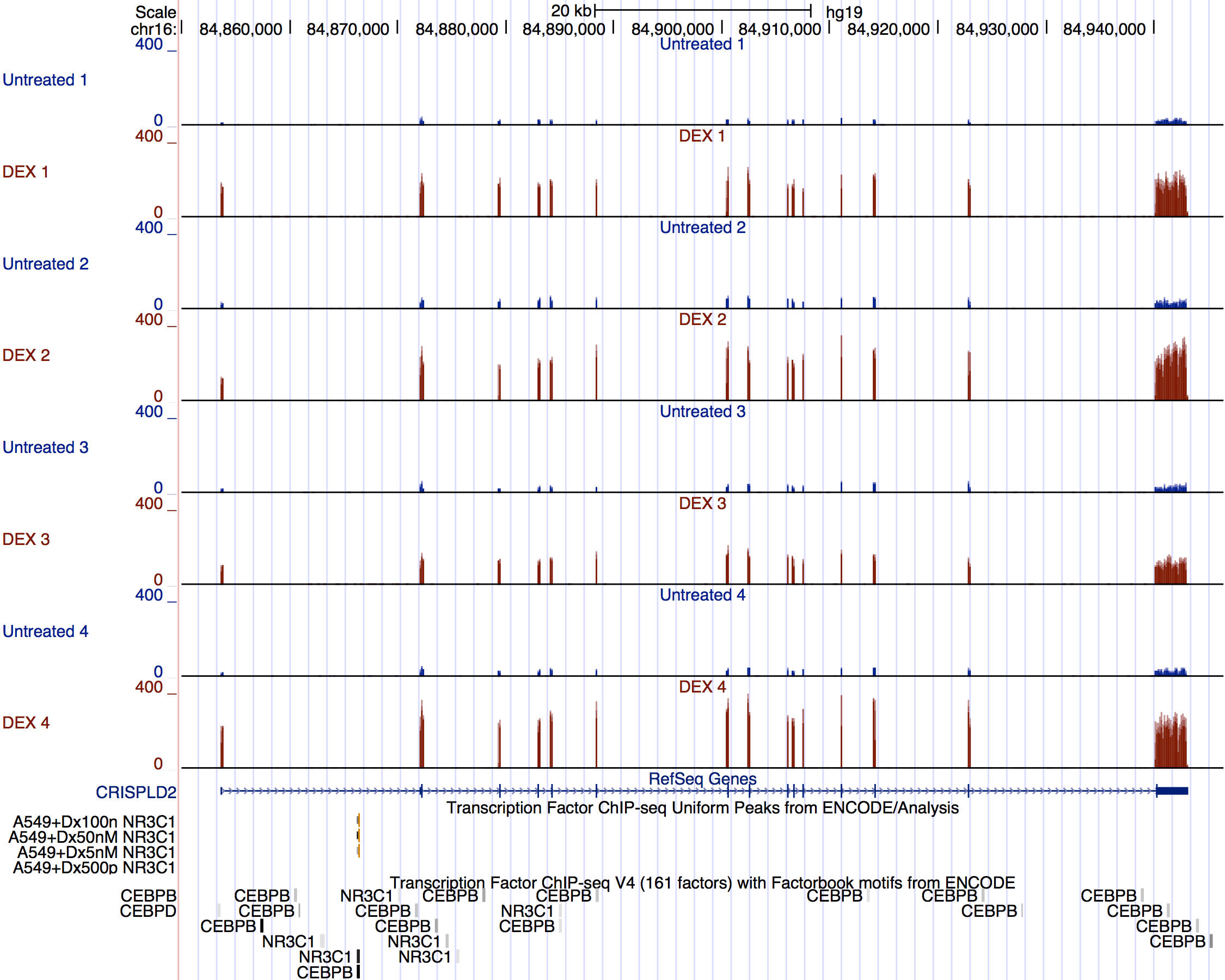

Supplement: Figure S8 — Raw RNA-Seq read plots for four ASM cell lines treated with DEX (red) or left untreated (blue) along the human (hg19) chromosome 16 region containing CRISPLD2 . Reads mapped to each exon of the RefSeq version of CRISPLD2. An increased number of mapped reads is observed in each sample after DEX treatment vs. when left untreated. Shown below the RefSeq gene track are ENCODE ChIP-Seq tracks: four are for sites found to bind the GR (official gene name NR3C1) in A549 pulmonary epithelial cells at various dosages of DEX (100 nM 50 nM, 5 nM, 500 pM) [12], and below these are Transcription Factor ChIP-Seq V4 results for GR, CEBPB, and CEBPD. Darker vertical lines represent binding sites with higher scores (i.e. detected more strongly). (TIFF) [file pone.0099625.s008.tif]

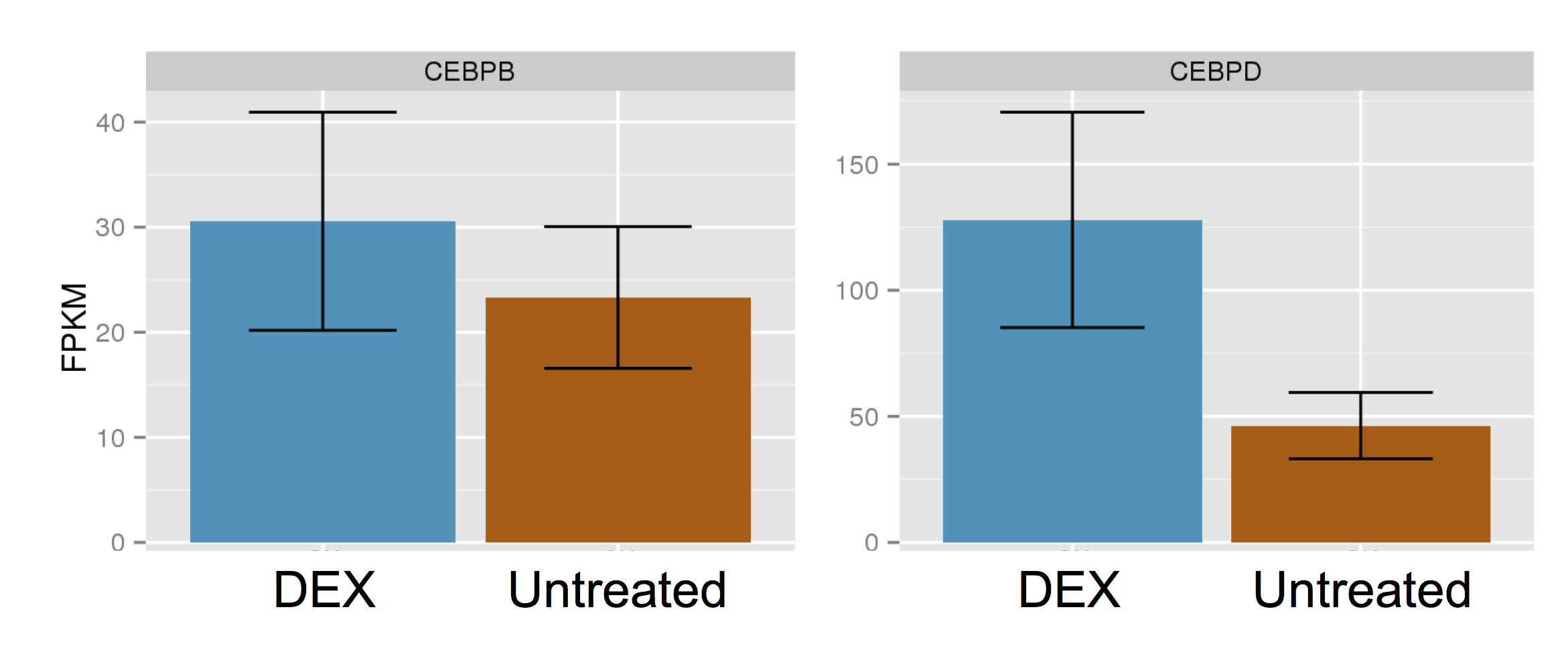

Supplement: Figure S9 — RNA-Seq results from four ASM cell lines treated with DEX expressed as FPKM for CEBPB and CEBPD by condition status (i.e. DEX vs. untreated) show the presence of both genes. DEX treatment did not significantly change the expression levels of CEBPB but did change the expression levels of CEBPD (Q-value 4.8E-04, Ln of fold-change 1.47). (TIFF) [file pone.0099625.s009.tif]

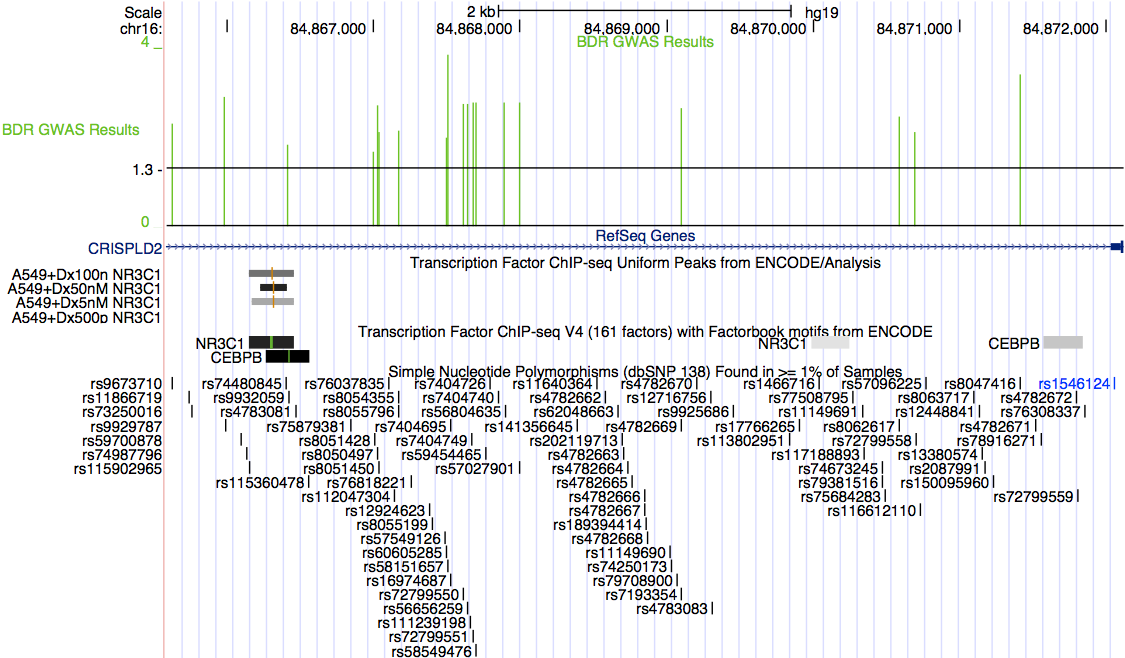

Supplement: Figure S10 — Region of CRISPLD2 where SNPs were most strongly associated with bronchodilator response (BDR) along with GR- and CEBPB-binding sites identified by ENCODE Transcription Factor ChIP-Seq V4 results. The x-axis denotes position along Chromosome 16 in hg19 genome build coordinates. The vertical axis of the BDR GWAS Results denotes –Log10(P-values), and the horizontal line at 1.3 represents a nominal significance threshold of P-value = 0.05. Shown below the RefSeq gene track are ENCODE ChIP-Seq tracks for sites found to bind the GR (official gene name NR3C1) in A549 pulmonary epithelial cells at various dosages of DEX (100 nM 50 nM, 5 nM, 500 pM) [12], and below these are Transcription Factor ChIP-Seq V4 results for GR, CEBPB, and CEBPD. Darker ChIP-Seq regions represent binding sites with higher scores (i.e. detected more strongly). (TIFF) [file pone.0099625.s010.tif]

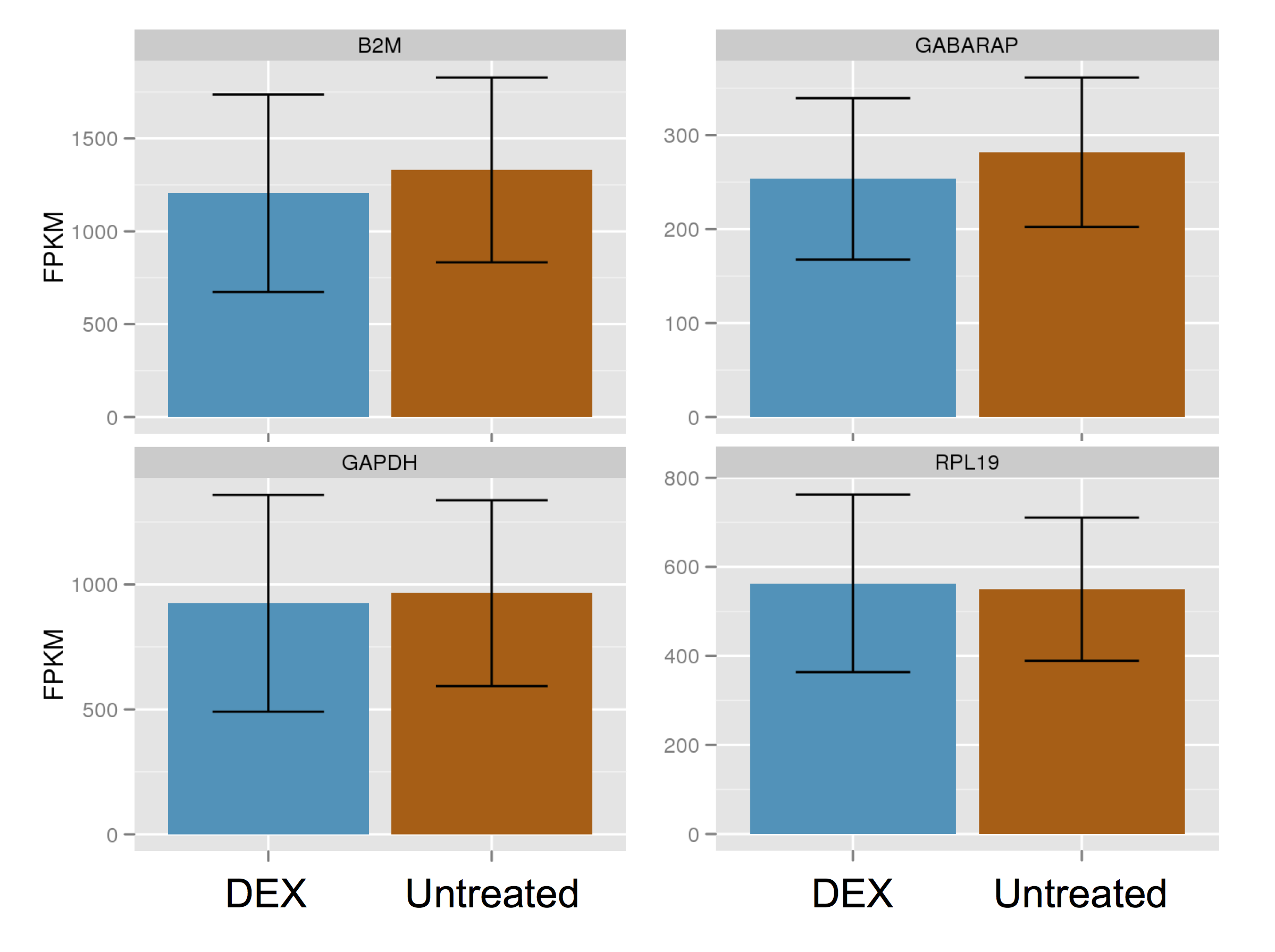

Supplement: Figure S11 — RNA-Seq results from four ASM cell lines treated with DEX expressed as FPKM for four housekeeping genes (i.e. B2M , GABARAP , GAPDH , RPL19 ) by condition status (i.e. DEX vs. untreated) show high levels of expression for each gene that did not significantly differ with DEX treatment. (TIFF) [file pone.0099625.s011.tif]
