# Supplementary material for: RNA-Seq Transcriptome Profiling Identifies CRISPLD2 as a Glucocorticoid Responsive Gene that Modulates Cytokine Function in Airway Smooth Muscle Cells
Source: PLoS One. 2014 Jun 13;9(6):e99625. doi: 10.1371/journal.pone.0099625 (PMC4057123; doi:10.1371/journal.pone.0099625)
Supplement: Table S1 — Number of various read types per sample in millions. Values in parenthesis for unmapped and mapped reads correspond to percentages of total reads per sample, while values in parenthesis for all other entries correspond to percentages of total mapped reads per sample. (DOCX) [file pone.0099625.s012.docx]

|  | Control.1 | Dex.1 | Control.2 | Dex.2 | Control.3 | Dex.3 | Control.4 | Dex.4 |
| --- | --- | --- | --- | --- | --- | --- | --- | --- |
| Total | 48.2 | 44.2 | 59.0 | 62.7 | 60.0 | 71.3 | 58.4 | 67.2 |
| Unmapped | 7.55 (15.66) | 7.66 (17.32) | 9.23 (15.66) | 1.05 (16.78) | 10.8 (18.06) | 12.3 (17.19) | 9.56 (16.37) | 10.8 (16.08) |
| Mapped | 40.6 (84.34) | 36.5 (82.68) | 49.7 (84.34) | 5.22 (83.22) | 49.2 (81.94) | 59.0 (82.81) | 48.9 (83.63) | 56.4 (83.92) |
| Forward strand | 20.3 (50.00) | 18.3 (50.01) | 24.9 (50.01) | 2.61 (50.02) | 24.6 (50.02) | 29.5 (50.02) | 24.4 (50.02) | 28.2 (50.02) |
| Reverse strand | 20.3 (50.00) | 18.3 (49.99) | 24.9 (49.99) | 2.61 (49.98) | 24.6 (49.98) | 29.5 (49.98) | 24.4 (49.98) | 28.2 (49.98) |
| Proper-pairs | 30.8 (75.86) | 27.0 (73.79) | 36.0 (72.33) | 2.18 (41.78) | 32.6 (66.35) | 43.4 (73.59) | 28.0 (57.39) | 30.8 (54.60) |
| Both pairs mapped | 38.8 (95.53) | 34.8 (95.11) | 47.4 (95.27) | 2.75 (52.80) | 44.0 (89.40) | 55.4 (93.90) | 35.2 (71.93) | 38.7 (68.56) |
| Read 1 | 20.5 (50.34) | 18.4 (50.48) | 24.9 (50.13) | 1.47 (28.12) | 23.4 (47.62) | 29.5 (49.97) | 18.7 (38.25) | 20.6 (36.52) |
| Read 2 | 20.2 (49.66) | 18.1 (49.52) | 24.8 (49.87) | 3.75 (71.88) | 25.8 (52.38) | 29.5 (50.03) | 30.2 (61.75) | 35.8 (63.48) |
| Singletons | 1.82 (4.47) | 1.79 (4.89) | 2.35 (4.73) | 2.46 (47.20) | 5.21 (10.60) | 3.60 (6.10) | 13.7 (28.07) | 17.7 (31.44) |
| Junction Spanning | 10.9 (26.93) | 9.69 (26.51) | 1.34 (26.88) | 1.31 (25.10) | 1.32 (26.82) | 1.56 (26.43) | 13.0 (26.64) | 14.8 (26.16) |
