# Supplementary material for: RNA-Seq Transcriptome Profiling Identifies CRISPLD2 as a Glucocorticoid Responsive Gene that Modulates Cytokine Function in Airway Smooth Muscle Cells
Source: PLoS One. 2014 Jun 13;9(6):e99625. doi: 10.1371/journal.pone.0099625 (PMC4057123; doi:10.1371/journal.pone.0099625)
Supplement: Table S2 — Percentage of mapped bases according to hg19 reference refFlat file base type. (DOCX) [file pone.0099625.s013.docx]

|  | Control.1 | Dex.1 | Control.2 | Dex.2 | Control.3 | Dex.3 | Control.4 | Dex.4 |
| --- | --- | --- | --- | --- | --- | --- | --- | --- |
| Coding | 65.70 | 64.50 | 66.10 | 61.40 | 65.50 | 65.20 | 65.40 | 65.00 |
| UTR | 32.50 | 34.00 | 32.40 | 37.30 | 32.90 | 33.50 | 33.20 | 33.60 |
| Intronic | 0.50 | 0.20 | 0.30 | 0.30 | 0.40 | 0.30 | 0.30 | 0.50 |
| Intergenic | 1.30 | 1.20 | 1.20 | 1.00 | 1.20 | 1.10 | 1.20 | 1.00 |
| mRNA | 98.20 | 98.60 | 98.50 | 98.70 | 98.40 | 98.60 | 98.60 | 98.50 |
