# Supplementary material for: RNA-Seq Transcriptome Profiling Identifies CRISPLD2 as a Glucocorticoid Responsive Gene that Modulates Cytokine Function in Airway Smooth Muscle Cells
Source: PLoS One. 2014 Jun 13;9(6):e99625. doi: 10.1371/journal.pone.0099625 (PMC4057123; doi:10.1371/journal.pone.0099625)
Supplement: Table S3 — Significantly differentially expressed genes. (DOCX) [file pone.0099625.s014.docx]

| Gene | Dex FPKM | Untreated FPKM | Ln[Fold Change] | Test Statistic | P-Value | Q-Value | Locus |
| --- | --- | --- | --- | --- | --- | --- | --- |
| *C7* | 38.41 | 3.76 | -3.35 | 8.74 | 0 | 0 | chr5:40909598-40983042 |
| *CCDC69* | 47.39 | 6.24 | -2.92 | 8.61 | 0 | 0 | chr5:150560612-150603654 |
| *DUSP1* | 144.96 | 18.26 | -2.99 | 8.99 | 0 | 0 | chr5:172195092-172198203 |
| *FKBP5* | 53.05 | 3.43 | -3.95 | 10.52 | 0 | 0 | chr6:35541361-35704724 |
| *GPX3* | 613.37 | 45.18 | -3.76 | 9.19 | 0 | 0 | chr5:150399998-150408554 |
| *KLF15* | 20.46 | 0.86 | -4.58 | 8.42 | 0 | 0 | chr3:126061477-126076236 |
| *MAOA* | 43.24 | 4.41 | -3.29 | 9.63 | 0 | 0 | chrX:43515408-43606068 |
| *SAMHD1* | 245.78 | 17.27 | -3.83 | 9.87 | 0 | 0 | chr20:35504569-35580246 |
| *SERPINA3* | 139.89 | 13.78 | -3.34 | 9.25 | 0 | 0 | chr14:95078713-95090390 |
| *SPARCL1* | 27.88 | 1.07 | -4.70 | 9.84 | 0 | 0 | chr4:88394487-88450655 |
| *C13orf15* | 96.35 | 10.00 | -3.27 | 8.18 | 2.2E-16 | 2.5E-13 | chr13:42031541-42045013 |
| *TSC22D3* | 93.26 | 9.69 | -3.27 | 8.23 | 2.2E-16 | 2.5E-13 | chrX:106956451-107019017 |
| *CRISPLD2* | 51.17 | 7.89 | -2.70 | 8.08 | 6.7E-16 | 6.9E-13 | chr16:84853586-84943116 |
| *PER1* | 13.69 | 1.49 | -3.20 | 7.86 | 3.8E-15 | 3.6E-12 | chr17:8043787-8055753 |
| *KCTD12* | 5.43 | 31.25 | 2.52 | -7.71 | 1.2E-14 | 1.1E-11 | chr13:77454303-77460540 |
| *ERRFI1* | 72.70 | 13.48 | -2.43 | 7.41 | 1.3E-13 | 1.1E-10 | chr1:8071778-8086393 |
| *STEAP4* | 6.35 | 0.20 | -4.99 | 7.19 | 6.7E-13 | 5.3E-10 | chr7:87905743-87936228 |
| *MT2A* | 561.55 | 119.33 | -2.23 | 6.96 | 3.4E-12 | 2.6E-09 | chr16:56642477-56643409 |
| *SOX4* | 4.00 | 21.26 | 2.41 | -6.85 | 7.2E-12 | 5.1E-09 | chr6:21593971-21598849 |
| *METTL7A* | 83.26 | 17.33 | -2.26 | 6.80 | 1.0E-11 | 6.9E-09 | chr12:51318533-51326300 |
| *IRS2* | 24.99 | 5.55 | -2.17 | 6.67 | 2.6E-11 | 1.7E-08 | chr13:110406183-110438914 |
| *RASL11A* | 53.65 | 9.85 | -2.45 | 6.62 | 3.7E-11 | 2.2E-08 | chr13:27844463-27847827 |
| *FGD4* | 9.57 | 2.05 | -2.23 | 6.53 | 6.4E-11 | 3.6E-08 | chr12:32655040-32798984 |
| *INHBB* | 11.18 | 0.97 | -3.53 | 6.54 | 6.3E-11 | 3.6E-08 | chr2:121103718-121109383 |
| *NNMT* | 357.28 | 77.94 | -2.20 | 6.48 | 9.4E-11 | 5.1E-08 | chr11:114166534-114183238 |
| *KCNK6* | 15.76 | 2.21 | -2.83 | 6.45 | 1.1E-10 | 5.6E-08 | chr19:38810483-38819649 |
| *GLUL* | 345.16 | 43.70 | -2.98 | 6.45 | 1.2E-10 | 5.8E-08 | chr1:182350838-182361341 |
| *KLF9* | 22.89 | 5.41 | -2.08 | 6.37 | 1.9E-10 | 9.1E-08 | chr9:72999512-73029573 |
| *LOC100507632* | 50.97 | 11.98 | -2.09 | 6.36 | 2.0E-10 | 9.3E-08 | chr8:57430877-57472382 |
| *ITGA10* | 4.21 | 0.27 | -3.95 | 6.31 | 2.7E-10 | 1.2E-07 | chr1:145524989-145543868 |
| *CORO6* | 38.56 | 7.08 | -2.44 | 6.24 | 4.4E-10 | 1.9E-07 | chr17:27920526-27948441 |
| *ADRA1B* | 9.92 | 0.65 | -3.93 | 6.16 | 7.3E-10 | 3.1E-07 | chr5:159343739-159400017 |
| *IMPA2* | 32.53 | 6.03 | -2.43 | 6.12 | 9.4E-10 | 3.8E-07 | chr18:11981426-12030885 |
| *ZBTB16* | 15.28 | 0.08 | -7.56 | 6.07 | 1.3E-09 | 5.2E-07 | chr11:113930430-114121397 |
| *FOXO1* | 8.44 | 1.72 | -2.30 | 6.04 | 1.5E-09 | 5.9E-07 | chr13:41129800-41240734 |
| *ADAMTS5* | 27.12 | 6.31 | -2.10 | 6.02 | 1.7E-09 | 6.4E-07 | chr21:28290230-28339439 |
| *PDK4* | 17.87 | 2.97 | -2.59 | 6.02 | 1.8E-09 | 6.4E-07 | chr7:95212808-95225925 |
| *ENDOD1* | 29.12 | 7.50 | -1.96 | 6.00 | 2.0E-09 | 7.0E-07 | chr11:94822973-94865815 |
| *SORT1* | 54.34 | 11.74 | -2.21 | 5.94 | 2.9E-09 | 9.9E-07 | chr1:109852187-109940563 |
| *CACNB2* | 10.18 | 0.99 | -3.36 | 5.92 | 3.3E-09 | 1.1E-06 | chr10:18429605-18830688 |
| *ADARB1* | 26.70 | 5.18 | -2.37 | 5.90 | 3.6E-09 | 1.2E-06 | chr21:46494492-46646478 |
| *NEXN* | 111.76 | 27.65 | -2.02 | 5.90 | 3.7E-09 | 1.2E-06 | chr1:78354199-78409578 |
| *NAV3* | 4.71 | 0.99 | -2.25 | 5.89 | 3.8E-09 | 1.2E-06 | chr12:78225068-78606790 |
| *DCXR* | 62.27 | 12.13 | -2.36 | 5.83 | 5.7E-09 | 1.7E-06 | chr17:79993756-79995573 |
| *STC1* | 17.81 | 3.33 | -2.42 | 5.83 | 5.6E-09 | 1.7E-06 | chr8:23699433-23712320 |
| *TRNP1* | 41.28 | 10.39 | -1.99 | 5.80 | 6.7E-09 | 2.0E-06 | chr1:27320194-27327377 |
| *NOV* | 2.10 | 12.69 | 2.60 | -5.78 | 7.4E-09 | 2.1E-06 | chr8:120428551-120436678 |
| *IGFBP2* | 120.50 | 27.25 | -2.14 | 5.76 | 8.3E-09 | 2.3E-06 | chr2:217498126-217529158 |
| *MAMDC2* | 15.17 | 3.23 | -2.23 | 5.71 | 1.1E-08 | 3.1E-06 | chr9:72658496-72873790 |
| *SUN2* | 74.85 | 19.18 | -1.96 | 5.71 | 1.1E-08 | 3.1E-06 | chr22:39130718-39152024 |
| *FSTL3* | 54.78 | 14.73 | -1.89 | 5.70 | 1.2E-08 | 3.2E-06 | chr19:676388-683392 |
| *LRRC17* | 8.48 | 40.86 | 2.27 | -5.67 | 1.5E-08 | 3.8E-06 | chr7:102453307-102715288 |
| *TBX18* | 9.65 | 34.23 | 1.83 | -5.65 | 1.6E-08 | 4.2E-06 | chr6:85444156-85473899 |
| *SCD* | 4.33 | 17.17 | 1.99 | -5.64 | 1.7E-08 | 4.3E-06 | chr10:102106771-102124588 |
| *BDKRB2* | 24.15 | 91.08 | 1.92 | -5.62 | 1.9E-08 | 4.7E-06 | chr14:96671134-96710666 |
| *PTX3* | 389.65 | 83.36 | -2.22 | 5.61 | 2.0E-08 | 4.8E-06 | chr3:156977531-157221415 |
| *WNT2* | 1.04 | 9.36 | 3.17 | -5.61 | 2.1E-08 | 4.9E-06 | chr7:116916685-116963343 |
| *GPRC5B* | 54.74 | 14.75 | -1.89 | 5.57 | 2.5E-08 | 5.8E-06 | chr16:19870292-19896151 |
| *HSPA2* | 13.52 | 2.34 | -2.53 | 5.55 | 2.9E-08 | 6.7E-06 | chr14:65007185-65009954 |
| *FAM107A* | 3.96 | 0.16 | -4.67 | 5.53 | 3.1E-08 | 7.0E-06 | chr3:58549844-58563491 |
| *TJP2* | 86.27 | 21.72 | -1.99 | 5.54 | 3.1E-08 | 7.0E-06 | chr9:71736179-71870124 |
| *PPP1R14A* | 32.96 | 3.76 | -3.13 | 5.53 | 3.3E-08 | 7.1E-06 | chr19:38741876-38747231 |
| *SLC7A14* | 0.24 | 1.87 | 2.98 | -5.48 | 4.1E-08 | 8.9E-06 | chr3:170177341-170303863 |
| *FER1L6* | 0.33 | 3.05 | 3.20 | -5.48 | 4.3E-08 | 9.1E-06 | chr8:124864226-125132302 |
| *MTSS1* | 22.92 | 5.96 | -1.94 | 5.39 | 6.9E-08 | 1.4E-05 | chr8:125563027-125740730 |
| *CLDN11* | 26.48 | 89.44 | 1.76 | -5.34 | 9.3E-08 | 1.9E-05 | chr3:170136652-170152479 |
| *VCAM1* | 1.00 | 11.98 | 3.58 | -5.33 | 1.0E-07 | 2.0E-05 | chr1:101185195-101204601 |
| *CYR61* | 163.09 | 45.48 | -1.84 | 5.32 | 1.1E-07 | 2.1E-05 | chr1:86046443-86049648 |
| *ADAMTS14* | 2.03 | 10.55 | 2.38 | -5.31 | 1.1E-07 | 2.1E-05 | chr10:72432558-72522195 |
| *USP53* | 78.27 | 17.52 | -2.16 | 5.30 | 1.1E-07 | 2.2E-05 | chr4:120133781-120216673 |
| *GPM6B* | 34.28 | 4.98 | -2.78 | 5.26 | 1.5E-07 | 2.8E-05 | chrX:13789061-13956831 |
| *ADAMTS1* | 476.33 | 96.16 | -2.31 | 5.18 | 2.2E-07 | 4.1E-05 | chr21:28208605-28217728 |
| *TRPC6* | 8.64 | 2.25 | -1.94 | 5.14 | 2.8E-07 | 5.2E-05 | chr11:101322294-101454659 |
| *RASL11B* | 5.56 | 0.34 | -4.03 | 5.12 | 3.1E-07 | 5.6E-05 | chr4:53728494-53733002 |
| *LMCD1* | 147.37 | 47.59 | -1.63 | 5.03 | 4.8E-07 | 8.6E-05 | chr3:8543510-8609806 |
| *MMD* | 41.54 | 13.56 | -1.62 | 5.02 | 5.3E-07 | 9.3E-05 | chr17:53469973-53499341 |
| *CCKAR* | 11.48 | 49.63 | 2.11 | -5.01 | 5.5E-07 | 9.7E-05 | chr4:26483017-26492042 |
| *ACSL1* | 24.80 | 8.13 | -1.61 | 5.00 | 5.8E-07 | 9.9E-05 | chr4:185676748-185747215 |
| *PKDCC* | 41.65 | 13.22 | -1.66 | 5.00 | 5.8E-07 | 9.9E-05 | chr2:42275160-42285668 |
| *STEAP1* | 94.51 | 31.97 | -1.56 | 4.94 | 7.6E-07 | 1.3E-04 | chr7:89783688-89794141 |
| *ACSS1* | 11.88 | 3.09 | -1.94 | 4.94 | 8.0E-07 | 1.3E-04 | chr20:24986865-25038818 |
| *TIMP4* | 9.70 | 1.34 | -2.85 | 4.93 | 8.2E-07 | 1.4E-04 | chr3:12045861-12233532 |
| *CREB5* | 1.29 | 5.22 | 2.01 | -4.91 | 9.0E-07 | 1.5E-04 | chr7:28338939-28865511 |
| *COL11A1* | 3.34 | 0.65 | -2.36 | 4.91 | 9.2E-07 | 1.5E-04 | chr1:103342022-103574052 |
| *TNFAIP6* | 2.94 | 16.46 | 2.49 | -4.91 | 9.3E-07 | 1.5E-04 | chr2:152214105-152236562 |
| *CRABP2* | 35.22 | 108.32 | 1.62 | -4.88 | 1.1E-06 | 1.7E-04 | chr1:156669399-156675608 |
| *PLXNA4* | 5.35 | 0.69 | -2.96 | 4.88 | 1.1E-06 | 1.7E-04 | chr7:131808090-132333447 |
| *MT1X* | 34.21 | 3.85 | -3.15 | 4.85 | 1.3E-06 | 1.9E-04 | chr16:56716381-56718108 |
| *APCDD1* | 40.74 | 11.84 | -1.78 | 4.78 | 1.8E-06 | 2.7E-04 | chr18:10454624-10488698 |
| *DNAJB4* | 36.69 | 12.72 | -1.53 | 4.75 | 2.0E-06 | 3.0E-04 | chr1:78470635-78482995 |
| *ACTG2* | 65.47 | 16.50 | -1.99 | 4.73 | 2.2E-06 | 3.3E-04 | chr2:74120092-74146780 |
| *GGT5* | 15.93 | 3.04 | -2.39 | 4.73 | 2.2E-06 | 3.3E-04 | chr22:24615621-24641110 |
| *IGDCC4* | 0.93 | 4.26 | 2.20 | -4.69 | 2.8E-06 | 4.0E-04 | chr15:65673824-65715410 |
| *LEP* | 2.60 | 0.29 | -3.17 | 4.69 | 2.8E-06 | 4.0E-04 | chr7:127881330-127897682 |
| *TMEM35* | 8.20 | 26.27 | 1.68 | -4.68 | 2.8E-06 | 4.0E-04 | chrX:100333835-100351355 |
| *CD302* | 63.78 | 20.02 | -1.67 | 4.67 | 2.9E-06 | 4.1E-04 | chr2:160625138-160761267 |
| *MT1E* | 56.37 | 12.19 | -2.21 | 4.67 | 3.1E-06 | 4.3E-04 | chr16:56659584-56661024 |
| *AOX1* | 55.39 | 18.74 | -1.56 | 4.64 | 3.4E-06 | 4.7E-04 | chr2:201450730-201536217 |
| *GDF15* | 8.71 | 32.47 | 1.90 | -4.64 | 3.4E-06 | 4.7E-04 | chr19:18496967-18499986 |
| *CEBPD* | 127.92 | 46.33 | -1.47 | 4.64 | 3.6E-06 | 4.8E-04 | chr8:48649475-48650726 |
| *DAAM2* | 27.77 | 8.55 | -1.70 | 4.62 | 3.8E-06 | 5.1E-04 | chr6:39760158-39902290 |
| *TSLP* | 0.63 | 4.64 | 2.88 | -4.62 | 3.8E-06 | 5.1E-04 | chr5:110405777-110413722 |
| *TEX2* | 44.43 | 15.85 | -1.49 | 4.61 | 4.0E-06 | 5.3E-04 | chr17:62224794-62340653 |
| *ADAM12* | 3.58 | 12.58 | 1.81 | -4.60 | 4.2E-06 | 5.4E-04 | chr10:127702901-128077127 |
| *AQP3* | 0.84 | 6.28 | 2.91 | -4.60 | 4.2E-06 | 5.4E-04 | chr9:33441151-33447631 |
| *FST* | 25.46 | 77.46 | 1.61 | -4.60 | 4.2E-06 | 5.4E-04 | chr5:52776263-52782304 |
| *TCEAL4* | 155.16 | 53.59 | -1.53 | 4.57 | 4.8E-06 | 6.1E-04 | chrX:102840418-102842655 |
| *PER3* | 0.54 | 2.67 | 2.32 | -4.53 | 5.9E-06 | 7.4E-04 | chr1:7844762-7905237 |
| *PRSS35* | 1.33 | 9.61 | 2.85 | -4.50 | 6.7E-06 | 8.3E-04 | chr6:84222193-84235421 |
| *SAT1* | 219.58 | 80.68 | -1.44 | 4.47 | 7.7E-06 | 9.5E-04 | chrX:23801274-23804327 |
| *PHC2* | 75.17 | 27.40 | -1.46 | 4.43 | 9.5E-06 | 1.1E-03 | chr1:33789223-33841194 |
| *SPON1* | 23.51 | 6.80 | -1.79 | 4.43 | 9.5E-06 | 1.1E-03 | chr11:13984183-14289679 |
| *KLF5* | 3.28 | 0.55 | -2.59 | 4.41 | 1.0E-05 | 1.2E-03 | chr13:73633141-73651676 |
| *AFAP1L1* | 7.65 | 2.32 | -1.72 | 4.40 | 1.1E-05 | 1.2E-03 | chr5:148651400-148721367 |
| *NKD1* | 1.42 | 0.26 | -2.48 | 4.41 | 1.1E-05 | 1.2E-03 | chr16:50582240-50674771 |
| *PIK3R1* | 31.02 | 10.40 | -1.58 | 4.40 | 1.1E-05 | 1.2E-03 | chr5:67511583-67597649 |
| *CDC42EP3* | 18.93 | 6.87 | -1.46 | 4.39 | 1.1E-05 | 1.3E-03 | chr2:37870742-37899326 |
| *CTPS* | 13.87 | 4.70 | -1.56 | 4.38 | 1.2E-05 | 1.4E-03 | chr1:41445006-41478235 |
| *PIEZO2* | 0.18 | 1.17 | 2.69 | -4.38 | 1.2E-05 | 1.4E-03 | chr18:10670243-11148761 |
| *INPP5A* | 33.07 | 12.21 | -1.44 | 4.36 | 1.3E-05 | 1.5E-03 | chr10:134351352-134596984 |
| *PRODH* | 2.14 | 0.03 | -6.18 | 4.36 | 1.3E-05 | 1.5E-03 | chr22:18900286-18924066 |
| *TNFRSF11B* | 71.02 | 207.57 | 1.55 | -4.34 | 1.4E-05 | 1.6E-03 | chr8:119935795-119964383 |
| *FBN2* | 101.92 | 22.90 | -2.15 | 4.33 | 1.5E-05 | 1.6E-03 | chr5:127593600-127873735 |
| *PXDC1* | 50.26 | 19.22 | -1.39 | 4.33 | 1.5E-05 | 1.7E-03 | chr6:3722835-3752246 |
| *RND2* | 0.75 | 3.62 | 2.27 | -4.31 | 1.6E-05 | 1.8E-03 | chr17:41177257-41184058 |
| *NID1* | 106.20 | 35.97 | -1.56 | 4.30 | 1.7E-05 | 1.8E-03 | chr1:236139131-236228481 |
| *ALPK3* | 0.50 | 0.06 | -3.14 | 4.28 | 1.8E-05 | 1.9E-03 | chr15:85359910-85416713 |
| *NR4A3* | 2.79 | 0.60 | -2.22 | 4.28 | 1.8E-05 | 1.9E-03 | chr9:102584136-102629173 |
| *HPS5* | 12.64 | 4.27 | -1.56 | 4.28 | 1.9E-05 | 2.0E-03 | chr11:18300216-18343721 |
| *PDPN* | 26.76 | 7.50 | -1.84 | 4.28 | 1.9E-05 | 2.0E-03 | chr1:13910251-13944452 |
| *EPHB6* | 37.07 | 14.16 | -1.39 | 4.25 | 2.1E-05 | 2.2E-03 | chr7:142552791-142568847 |
| *ATF3* | 13.16 | 3.25 | -2.02 | 4.25 | 2.1E-05 | 2.2E-03 | chr1:212738675-212794119 |
| *OXTR* | 8.83 | 2.29 | -1.95 | 4.25 | 2.2E-05 | 2.2E-03 | chr3:8792094-8811300 |
| *RAB11FIP1* | 15.90 | 5.43 | -1.55 | 4.23 | 2.3E-05 | 2.4E-03 | chr8:37716464-37757015 |
| *CITED2* | 281.66 | 91.69 | -1.62 | 4.22 | 2.5E-05 | 2.5E-03 | chr6:139693396-139695785 |
| *RAPGEF5* | 1.01 | 0.14 | -2.83 | 4.22 | 2.5E-05 | 2.5E-03 | chr7:22157907-22396533 |
| *CDH6* | 5.86 | 15.82 | 1.43 | -4.21 | 2.5E-05 | 2.5E-03 | chr5:31193761-31329253 |
| *COL4A1* | 22.61 | 8.87 | -1.35 | 4.18 | 2.9E-05 | 2.8E-03 | chr13:110801309-110959496 |
| *NEGR1* | 14.47 | 5.47 | -1.40 | 4.18 | 2.9E-05 | 2.8E-03 | chr1:71868624-72748277 |
| *LIF* | 0.58 | 3.08 | 2.41 | -4.17 | 3.0E-05 | 2.9E-03 | chr22:30636441-30642796 |
| *ITGA11* | 5.02 | 14.70 | 1.55 | -4.17 | 3.1E-05 | 3.0E-03 | chr15:68594041-68724492 |
| *PRKAG2* | 26.62 | 9.40 | -1.50 | 4.16 | 3.2E-05 | 3.0E-03 | chr7:151253200-151576308 |
| *ADCY1* | 0.43 | 2.07 | 2.27 | -4.15 | 3.3E-05 | 3.1E-03 | chr7:45614124-45762714 |
| *SIPA1L2* | 0.21 | 1.33 | 2.67 | -4.14 | 3.5E-05 | 3.2E-03 | chr1:232533711-232651243 |
| *PHGDH* | 16.13 | 41.76 | 1.37 | -4.12 | 3.8E-05 | 3.6E-03 | chr1:120254418-120286849 |
| *AMOT* | 0.82 | 3.82 | 2.22 | -4.11 | 3.9E-05 | 3.6E-03 | chrX:112018104-112084043 |
| *HMOX1* | 42.56 | 105.90 | 1.32 | -4.11 | 3.9E-05 | 3.6E-03 | chr22:35777059-35790207 |
| *SLC16A12* | 0.76 | 0.05 | -3.88 | 4.11 | 3.9E-05 | 3.6E-03 | chr10:91190050-91295313 |
| *SMARCD2* | 51.94 | 21.09 | -1.30 | 4.11 | 3.9E-05 | 3.6E-03 | chr17:61909440-61920351 |
| *NEDD9* | 27.21 | 8.36 | -1.70 | 4.11 | 4.0E-05 | 3.6E-03 | chr6:11183530-11382581 |
| *GALNTL2* | 186.27 | 62.66 | -1.57 | 4.10 | 4.0E-05 | 3.6E-03 | chr3:16216183-16271253 |
| *PGBD5* | 0.53 | 3.21 | 2.59 | -4.10 | 4.1E-05 | 3.6E-03 | chr1:230457391-230513391 |
| *CDON* | 0.93 | 3.10 | 1.73 | -4.06 | 4.9E-05 | 4.3E-03 | chr11:125826712-125933187 |
| *MXRA5* | 11.49 | 29.25 | 1.35 | -4.05 | 5.1E-05 | 4.5E-03 | chrX:3226608-3264684 |
| *MYADM* | 127.17 | 44.72 | -1.51 | 4.05 | 5.2E-05 | 4.5E-03 | chr19:54369610-54379689 |
| *FZD8* | 3.20 | 0.60 | -2.42 | 4.01 | 6.0E-05 | 5.2E-03 | chr10:35927176-35930362 |
| *PLA2G4A* | 0.46 | 2.71 | 2.56 | -4.00 | 6.2E-05 | 5.4E-03 | chr1:186798031-186958113 |
| *CILP* | 11.79 | 4.50 | -1.39 | 4.00 | 6.4E-05 | 5.5E-03 | chr15:65488336-65503840 |
| *GFPT2* | 21.31 | 8.43 | -1.34 | 3.99 | 6.5E-05 | 5.5E-03 | chr5:179727699-179780315 |
| *FAM171B* | 5.54 | 14.18 | 1.36 | -3.99 | 6.7E-05 | 5.7E-03 | chr2:187558788-187628512 |
| *AHCTF1* | 7.51 | 3.00 | -1.33 | 3.98 | 6.8E-05 | 5.7E-03 | chr1:247002401-247094726 |
| *C3orf64* | 22.10 | 9.22 | -1.26 | 3.98 | 7.0E-05 | 5.8E-03 | chr3:69024367-69062774 |
| *CPA4* | 0.79 | 4.09 | 2.37 | -3.97 | 7.2E-05 | 5.9E-03 | chr7:129932973-129964020 |
| *SSH2* | 12.89 | 5.41 | -1.25 | 3.96 | 7.5E-05 | 6.2E-03 | chr17:27952964-28435470 |
| *C5orf62* | 31.85 | 13.18 | -1.27 | 3.96 | 7.5E-05 | 6.2E-03 | chr5:150157507-150176298 |
| *AIF1L* | 0.32 | 2.12 | 2.73 | -3.95 | 7.7E-05 | 6.2E-03 | chr9:133971862-133998539 |
| *DLL4* | 1.69 | 0.13 | -3.74 | 3.95 | 7.7E-05 | 6.2E-03 | chr15:41221530-41231258 |
| *GPR68* | 0.17 | 1.57 | 3.18 | -3.95 | 7.8E-05 | 6.2E-03 | chr14:91698875-91720224 |
| *HSD17B6* | 1.75 | 8.31 | 2.24 | -3.95 | 7.8E-05 | 6.2E-03 | chr12:57157107-57181574 |
| *LHFPL2* | 10.46 | 24.79 | 1.24 | -3.96 | 7.6E-05 | 6.2E-03 | chr5:77781037-77944648 |
| *SLC6A9* | 1.12 | 5.36 | 2.26 | -3.94 | 8.1E-05 | 6.4E-03 | chr1:44457279-44497134 |
| *C10orf10* | 650.61 | 206.68 | -1.65 | 3.93 | 8.5E-05 | 6.5E-03 | chr10:45455218-45490172 |
| *CHST7* | 14.65 | 5.13 | -1.51 | 3.93 | 8.5E-05 | 6.5E-03 | chrX:46433191-46457838 |
| *GCNT4* | 4.85 | 1.27 | -1.94 | 3.93 | 8.4E-05 | 6.5E-03 | chr5:74323288-74326724 |
| *SCARA5* | 6.97 | 2.11 | -1.72 | 3.93 | 8.5E-05 | 6.5E-03 | chr8:27727398-27850369 |
| *STON1* | 19.09 | 7.46 | -1.36 | 3.93 | 8.4E-05 | 6.5E-03 | chr2:48757063-49003656 |
| *SLC7A6* | 20.26 | 7.86 | -1.37 | 3.93 | 8.7E-05 | 6.6E-03 | chr16:68298418-68344868 |
| *C4orf46* | 3.74 | 10.15 | 1.44 | -3.91 | 9.1E-05 | 6.9E-03 | chr4:159587830-159593202 |
| *OLFML2A* | 5.43 | 14.27 | 1.40 | -3.91 | 9.1E-05 | 6.9E-03 | chr9:127539436-127577159 |
| *ABLIM1* | 6.94 | 2.66 | -1.38 | 3.91 | 9.3E-05 | 6.9E-03 | chr10:116190868-116444414 |
| *EPHB2* | 1.52 | 5.24 | 1.79 | -3.91 | 9.3E-05 | 6.9E-03 | chr1:23037330-23241823 |
| *NCOA3* | 11.87 | 4.52 | -1.39 | 3.91 | 9.3E-05 | 6.9E-03 | chr20:46130600-46285621 |
| *GXYLT2* | 6.17 | 18.41 | 1.58 | -3.90 | 9.8E-05 | 7.2E-03 | chr3:72937384-73024522 |
| *TGFBR2* | 180.20 | 65.23 | -1.47 | 3.89 | 9.8E-05 | 7.2E-03 | chr3:30647993-30735633 |
| *MOB3B* | 0.72 | 0.10 | -2.87 | 3.89 | 9.9E-05 | 7.2E-03 | chr9:27325206-27529850 |
| *COL4A4* | 1.34 | 0.33 | -2.00 | 3.89 | 1.0E-04 | 7.3E-03 | chr2:227867426-228029275 |
| *RNF144B* | 2.19 | 0.49 | -2.15 | 3.88 | 1.0E-04 | 7.4E-03 | chr6:18387580-18469105 |
| *FOXO3* | 39.42 | 16.30 | -1.27 | 3.88 | 1.0E-04 | 7.5E-03 | chr6:108881025-109005971 |
| *GCLM* | 26.98 | 11.39 | -1.24 | 3.88 | 1.1E-04 | 7.5E-03 | chr1:94352589-94375012 |
| *DAPK2* | 13.39 | 4.71 | -1.51 | 3.87 | 1.1E-04 | 7.6E-03 | chr15:64199234-64338521 |
| *SLC14A1* | 0.06 | 1.02 | 4.08 | -3.87 | 1.1E-04 | 7.6E-03 | chr18:43304091-43332485 |
| *MAP1LC3C* | 8.28 | 1.38 | -2.59 | 3.87 | 1.1E-04 | 7.7E-03 | chr1:242158791-242162385 |
| *LRRC15* | 0.60 | 2.86 | 2.24 | -3.87 | 1.1E-04 | 7.7E-03 | chr3:194075975-194090472 |
| *COL7A1* | 10.24 | 4.35 | -1.23 | 3.86 | 1.1E-04 | 7.8E-03 | chr3:48601505-48632593 |
| *CYTH3* | 53.05 | 22.48 | -1.24 | 3.86 | 1.2E-04 | 8.0E-03 | chr7:6201411-6312242 |
| *FIBIN* | 54.05 | 22.87 | -1.24 | 3.85 | 1.2E-04 | 8.3E-03 | chr11:27015627-27018632 |
| *DIO2* | 6.46 | 2.33 | -1.47 | 3.84 | 1.2E-04 | 8.4E-03 | chr14:80663867-80921810 |
| *ODZ4* | 1.09 | 2.94 | 1.43 | -3.83 | 1.3E-04 | 8.7E-03 | chr11:78364327-79151695 |
| *HIGD1A* | 45.42 | 19.19 | -1.24 | 3.83 | 1.3E-04 | 8.8E-03 | chr3:42824399-42846027 |
| *BDKRB1* | 3.49 | 13.29 | 1.93 | -3.83 | 1.3E-04 | 8.8E-03 | chr14:96722546-96731100 |
| *BMPER* | 2.70 | 7.38 | 1.45 | -3.80 | 1.4E-04 | 9.6E-03 | chr7:33944522-34195484 |
| *FAM46C* | 0.20 | 1.17 | 2.54 | -3.80 | 1.4E-04 | 9.6E-03 | chr1:118148603-118171011 |
| *CPM* | 7.74 | 2.82 | -1.46 | 3.80 | 1.5E-04 | 9.7E-03 | chr12:69244955-69357020 |
| *MARCKS* | 35.76 | 84.48 | 1.24 | -3.80 | 1.5E-04 | 9.7E-03 | chr6:114178526-114184652 |
| *NFIL3* | 42.94 | 18.46 | -1.22 | 3.80 | 1.5E-04 | 9.7E-03 | chr9:94171326-94186144 |
| *WARS* | 25.74 | 63.28 | 1.30 | -3.79 | 1.5E-04 | 9.7E-03 | chr14:100800124-100842680 |
| *RHOB* | 98.00 | 42.80 | -1.20 | 3.77 | 1.6E-04 | 0.010 | chr2:20646834-20649201 |
| *ARHGAP28* | 2.72 | 7.56 | 1.48 | -3.76 | 1.7E-04 | 0.011 | chr18:6834431-6915712 |
| *RCAN2* | 3.29 | 11.47 | 1.80 | -3.76 | 1.7E-04 | 0.011 | chr6:46188466-46459804 |
| *HSPB3* | 9.55 | 1.65 | -2.53 | 3.75 | 1.7E-04 | 0.011 | chr5:53751430-53752214 |
| *TMEM119* | 31.04 | 69.94 | 1.17 | -3.76 | 1.7E-04 | 0.011 | chr12:108983621-108991894 |
| *CD82* | 84.31 | 36.18 | -1.22 | 3.75 | 1.8E-04 | 0.011 | chr11:44587140-44641315 |
| *STARD7* | 68.18 | 29.14 | -1.23 | 3.75 | 1.8E-04 | 0.011 | chr2:96850602-96908362 |
| *WFDC1* | 13.22 | 3.79 | -1.80 | 3.75 | 1.8E-04 | 0.011 | chr16:84328400-84363450 |
| *ZCCHC5* | 1.97 | 0.24 | -3.04 | 3.75 | 1.8E-04 | 0.011 | chrX:77911565-77914825 |
| *RAP2B* | 2.55 | 6.40 | 1.33 | -3.74 | 1.8E-04 | 0.011 | chr3:152880028-152886263 |
| *ANXA4* | 137.24 | 59.80 | -1.20 | 3.72 | 2.0E-04 | 0.013 | chr2:69969126-70053596 |
| *CDKN2B* | 3.07 | 8.62 | 1.49 | -3.71 | 2.1E-04 | 0.013 | chr9:21994789-22121093 |
| *RAI2* | 30.37 | 11.08 | -1.45 | 3.70 | 2.2E-04 | 0.013 | chrX:17818168-17879457 |
| *KANK1* | 14.10 | 5.90 | -1.26 | 3.70 | 2.2E-04 | 0.013 | chr9:504702-746103 |
| *SCN7A* | 39.02 | 15.26 | -1.36 | 3.70 | 2.2E-04 | 0.013 | chr2:167260082-167343481 |
| *ABHD5* | 18.32 | 8.19 | -1.16 | 3.68 | 2.4E-04 | 0.014 | chr3:43732374-43764217 |
| *STOM* | 269.99 | 101.41 | -1.41 | 3.66 | 2.5E-04 | 0.015 | chr9:124101352-124132545 |
| *SYNPO2* | 59.81 | 17.52 | -1.77 | 3.66 | 2.5E-04 | 0.015 | chr4:119809995-119982402 |
| *LBH* | 305.38 | 110.41 | -1.47 | 3.65 | 2.6E-04 | 0.016 | chr2:30454396-30482899 |
| *RASD1* | 4.96 | 0.92 | -2.43 | 3.65 | 2.6E-04 | 0.016 | chr17:17397752-17399709 |
| *CALCOCO2* | 52.38 | 23.54 | -1.15 | 3.65 | 2.7E-04 | 0.016 | chr17:46908371-46942229 |
| *POTEE* | 13.30 | 5.23 | -1.35 | 3.64 | 2.7E-04 | 0.016 | chr2:131975923-132022416 |
| *C1QTNF1* | 23.55 | 9.82 | -1.26 | 3.64 | 2.7E-04 | 0.016 | chr17:77015290-77045870 |
| *MEST* | 5.62 | 20.36 | 1.86 | -3.64 | 2.8E-04 | 0.016 | chr7:130126015-130371406 |
| *IFIT1* | 2.57 | 8.57 | 1.74 | -3.63 | 2.8E-04 | 0.017 | chr10:91152321-91163744 |
| *SLC39A10* | 3.37 | 8.31 | 1.30 | -3.63 | 2.8E-04 | 0.017 | chr2:196521531-196602426 |
| *CADPS2* | 11.99 | 4.64 | -1.37 | 3.62 | 2.9E-04 | 0.017 | chr7:121958477-122526813 |
| *LAMA3* | 9.19 | 3.66 | -1.33 | 3.62 | 3.0E-04 | 0.017 | chr18:21269561-21535029 |
| *ZHX3* | 19.86 | 9.01 | -1.14 | 3.62 | 3.0E-04 | 0.017 | chr20:39807088-39928739 |
| *LGI3* | 0.63 | 0.01 | -5.44 | 3.60 | 3.1E-04 | 0.018 | chr8:22004342-22014344 |
| *ZCCHC6* | 13.98 | 6.28 | -1.15 | 3.60 | 3.2E-04 | 0.018 | chr9:88902647-88969402 |
| *TSPAN8* | 2.03 | 0.11 | -4.17 | 3.59 | 3.3E-04 | 0.019 | chr12:71518876-71551779 |
| *CORIN* | 2.02 | 0.52 | -1.97 | 3.59 | 3.3E-04 | 0.019 | chr4:47596017-47840059 |
| *ENPP1* | 5.05 | 2.03 | -1.32 | 3.59 | 3.3E-04 | 0.019 | chr6:132129155-132216295 |
| *EPHB3* | 2.51 | 6.86 | 1.45 | -3.59 | 3.3E-04 | 0.019 | chr3:184279586-184300196 |
| *GADD45B* | 19.89 | 7.34 | -1.44 | 3.59 | 3.3E-04 | 0.019 | chr19:2476122-2478257 |
| *SAMD4A* | 27.89 | 12.06 | -1.21 | 3.59 | 3.3E-04 | 0.019 | chr14:55034329-55260033 |
| *TUBB* | 16.10 | 36.25 | 1.17 | -3.59 | 3.3E-04 | 0.019 | chr6:30688156-30693195 |
| *RPS6KA5* | 0.28 | 1.66 | 2.55 | -3.58 | 3.4E-04 | 0.019 | chr14:91337166-91526993 |
| *LDB2* | 6.91 | 16.76 | 1.28 | -3.57 | 3.5E-04 | 0.019 | chr4:16503164-16900424 |
| *TUBA1A* | 145.51 | 343.69 | 1.24 | -3.57 | 3.5E-04 | 0.019 | chr12:49578582-49582861 |
| *LRRC16A* | 16.24 | 7.00 | -1.21 | 3.57 | 3.6E-04 | 0.020 | chr6:25279655-25620758 |
| *DHRS3* | 138.29 | 59.89 | -1.21 | 3.56 | 3.7E-04 | 0.020 | chr1:12627938-12677820 |
| *PREB* | 50.92 | 23.13 | -1.14 | 3.56 | 3.8E-04 | 0.020 | chr2:27346656-27357542 |
| *HMGB2* | 41.04 | 17.11 | -1.26 | 3.56 | 3.8E-04 | 0.020 | chr4:174252526-174255595 |
| *C14orf132* | 16.76 | 36.73 | 1.13 | -3.55 | 3.8E-04 | 0.020 | chr14:96505660-96560226 |
| *TMEM200A* | 3.71 | 8.63 | 1.22 | -3.54 | 3.9E-04 | 0.021 | chr6:130758261-130764210 |
| *NCKAP5* | 0.22 | 1.05 | 2.26 | -3.54 | 4.0E-04 | 0.021 | chr2:133429371-134326031 |
| *MAFF* | 18.26 | 7.44 | -1.30 | 3.53 | 4.1E-04 | 0.022 | chr22:38597938-38612517 |
| *NAMPT* | 21.51 | 9.94 | -1.11 | 3.52 | 4.3E-04 | 0.022 | chr7:105888731-105925638 |
| *TUBB3* | 7.43 | 20.54 | 1.47 | -3.52 | 4.3E-04 | 0.023 | chr16:89988416-90002505 |
| *PHF17* | 18.27 | 7.90 | -1.21 | 3.52 | 4.3E-04 | 0.023 | chr4:129730778-129796379 |
| *ARMC8* | 13.62 | 5.48 | -1.31 | 3.51 | 4.4E-04 | 0.023 | chr3:137906147-138048728 |
| *NEK10* | 0.23 | 1.41 | 2.63 | -3.51 | 4.4E-04 | 0.023 | chr3:27257096-27410912 |
| *NUAK1* | 3.86 | 1.50 | -1.36 | 3.52 | 4.4E-04 | 0.023 | chr12:106457124-106533811 |
| *PHLDA1* | 13.17 | 28.15 | 1.10 | -3.52 | 4.4E-04 | 0.023 | chr12:76419226-76425556 |
| *ARHGAP29* | 26.68 | 11.77 | -1.18 | 3.51 | 4.5E-04 | 0.023 | chr1:94634462-94703307 |
| *DDAH1* | 44.96 | 18.57 | -1.28 | 3.50 | 4.6E-04 | 0.023 | chr1:85784167-86044046 |
| *LOC148145* | 0.02 | 0.51 | 4.37 | -3.50 | 4.7E-04 | 0.024 | chr19:29456037-29460055 |
| *MICAL2* | 12.00 | 5.14 | -1.22 | 3.50 | 4.7E-04 | 0.024 | chr11:12132137-12285331 |
| *ANPEP* | 70.05 | 32.15 | -1.12 | 3.49 | 4.9E-04 | 0.024 | chr15:90328125-90358072 |
| *NYNRIN* | 5.76 | 12.42 | 1.11 | -3.49 | 4.8E-04 | 0.024 | chr14:24867991-24888494 |
| *RN5-8S1* | 4.57 | 77.42 | 4.08 | -3.49 | 4.9E-04 | 0.024 | chrUn_gl000220:155996-156152 |
| *SLC6A6* | 2.04 | 8.36 | 2.04 | -3.49 | 4.9E-04 | 0.024 | chr3:14444105-14583588 |
| *PNPLA2* | 65.95 | 31.15 | -1.08 | 3.48 | 4.9E-04 | 0.025 | chr11:818900-825571 |
| *KLF6* | 40.85 | 19.00 | -1.10 | 3.48 | 5.0E-04 | 0.025 | chr10:3818187-3827473 |
| *RHOJ* | 4.95 | 11.66 | 1.24 | -3.47 | 5.2E-04 | 0.026 | chr14:63671101-63760230 |
| *AASS* | 9.89 | 4.52 | -1.13 | 3.47 | 5.3E-04 | 0.026 | chr7:121713597-121784344 |
| *CBS* | 20.53 | 8.31 | -1.30 | 3.46 | 5.4E-04 | 0.026 | chr21:44473300-44496472 |
| *WASF3* | 14.70 | 6.85 | -1.10 | 3.46 | 5.4E-04 | 0.026 | chr13:27131839-27263082 |
| *FLRT3* | 0.60 | 3.07 | 2.36 | -3.44 | 5.8E-04 | 0.028 | chr20:13976145-16033841 |
| *LMOD1* | 104.56 | 46.06 | -1.18 | 3.44 | 5.8E-04 | 0.028 | chr1:201865583-201915716 |
| *RSPO1* | 14.07 | 5.40 | -1.38 | 3.43 | 6.0E-04 | 0.029 | chr1:38076950-38100595 |
| *TRIM45* | 0.69 | 2.67 | 1.96 | -3.43 | 6.0E-04 | 0.029 | chr1:117653676-117664411 |
| *AP1M1* | 59.10 | 27.69 | -1.09 | 3.43 | 6.1E-04 | 0.029 | chr19:16308664-16346156 |
| *FAM43A* | 15.33 | 6.62 | -1.21 | 3.43 | 6.1E-04 | 0.029 | chr3:194406621-194409766 |
| *FAM46B* | 2.44 | 0.49 | -2.32 | 3.43 | 6.1E-04 | 0.029 | chr1:27331510-27339333 |
| *CIT* | 0.71 | 2.04 | 1.52 | -3.41 | 6.4E-04 | 0.030 | chr12:120123594-120315095 |
| *PDE5A* | 25.67 | 61.64 | 1.26 | -3.41 | 6.4E-04 | 0.030 | chr4:120375937-120549981 |
| *SQRDL* | 86.31 | 41.15 | -1.07 | 3.41 | 6.4E-04 | 0.030 | chr15:45927255-45983479 |
| *RTKN2* | 0.24 | 1.03 | 2.08 | -3.41 | 6.5E-04 | 0.031 | chr10:63952952-64028466 |
| *ARSI* | 1.38 | 4.66 | 1.75 | -3.41 | 6.6E-04 | 0.031 | chr5:149675908-149682525 |
| *LINC00312* | 3.63 | 1.01 | -1.84 | 3.40 | 6.7E-04 | 0.031 | chr3:8613467-8616354 |
| *FZD5* | 0.61 | 0.11 | -2.50 | 3.40 | 6.8E-04 | 0.032 | chr2:208627309-208634143 |
| *SAP30* | 23.59 | 9.18 | -1.36 | 3.39 | 7.0E-04 | 0.033 | chr4:174292092-174298683 |
| *G0S2* | 0.62 | 4.15 | 2.75 | -3.38 | 7.4E-04 | 0.034 | chr1:209848669-209849735 |
| *VGLL3* | 70.61 | 29.39 | -1.26 | 3.38 | 7.3E-04 | 0.034 | chr3:86987122-87040257 |
| *ING2* | 22.08 | 8.64 | -1.35 | 3.37 | 7.6E-04 | 0.035 | chr4:184426219-184432249 |
| *VEGFA* | 12.99 | 29.77 | 1.20 | -3.37 | 7.6E-04 | 0.035 | chr6:43737945-43754223 |
| *TM4SF1* | 33.88 | 15.70 | -1.11 | 3.35 | 7.9E-04 | 0.036 | chr3:149086804-149095568 |
| *DUSP5* | 10.66 | 4.29 | -1.31 | 3.35 | 8.2E-04 | 0.037 | chr10:112257624-112271302 |
| *FADS3* | 61.17 | 29.54 | -1.05 | 3.32 | 9.1E-04 | 0.041 | chr11:61640997-61659006 |
| *CTGF* | 337.57 | 129.42 | -1.38 | 3.31 | 9.3E-04 | 0.042 | chr6:132269316-132272518 |
| *FAM46A* | 2.33 | 5.43 | 1.22 | -3.31 | 9.5E-04 | 0.043 | chr6:82455446-82462428 |
| *RWDD4* | 15.63 | 7.08 | -1.14 | 3.30 | 9.6E-04 | 0.043 | chr4:184560788-184580331 |
| *SEMA3A* | 2.81 | 6.36 | 1.18 | -3.30 | 9.7E-04 | 0.043 | chr7:83587658-83824217 |
| *JUN* | 28.75 | 62.24 | 1.11 | -3.29 | 9.8E-04 | 0.044 | chr1:59246462-59249785 |
| *SAMD12* | 0.18 | 0.78 | 2.09 | -3.29 | 9.8E-04 | 0.044 | chr8:119201694-119738306 |
| *KLHDC5* | 8.93 | 4.27 | -1.06 | 3.29 | 1.0E-03 | 0.045 | chr12:27933186-27955973 |
| *STK17B* | 15.31 | 7.27 | -1.07 | 3.29 | 1.0E-03 | 0.045 | chr2:196998306-197036336 |
| *CLIC2* | 3.60 | 8.81 | 1.29 | -3.28 | 1.0E-03 | 0.045 | chrX:154505499-154563986 |
| *REV3L* | 42.43 | 19.34 | -1.13 | 3.28 | 1.0E-03 | 0.045 | chr6:111620233-111804414 |
| *EBF1* | 9.67 | 4.56 | -1.09 | 3.27 | 1.1E-03 | 0.047 | chr5:158122922-158526788 |
| *SOX9* | 3.94 | 9.18 | 1.22 | -3.27 | 1.1E-03 | 0.047 | chr17:70117160-70122560 |
| *APBB2* | 15.37 | 7.24 | -1.09 | 3.26 | 1.1E-03 | 0.048 | chr4:40812043-41216635 |
| *HSD11B1* | 34.04 | 13.85 | -1.30 | 3.26 | 1.1E-03 | 0.048 | chr1:209859524-209908295 |
| *AXIN2* | 6.70 | 2.85 | -1.23 | 3.26 | 1.1E-03 | 0.048 | chr17:63524682-63557740 |
| *TXNRD1* | 388.43 | 135.73 | -1.52 | 3.26 | 1.1E-03 | 0.048 | chr12:104609558-104744062 |
| *GRAMD4* | 3.75 | 8.54 | 1.19 | -3.26 | 1.1E-03 | 0.049 | chr22:47022657-47075688 |
| *ABCA6* | 22.25 | 10.93 | -1.03 | 3.25 | 1.2E-03 | 0.049 | chr17:67074846-67138015 |
