# Supplementary material for: RNA-Seq Transcriptome Profiling Identifies CRISPLD2 as a Glucocorticoid Responsive Gene that Modulates Cytokine Function in Airway Smooth Muscle Cells
Source: PLoS One. 2014 Jun 13;9(6):e99625. doi: 10.1371/journal.pone.0099625 (PMC4057123; doi:10.1371/journal.pone.0099625)
Supplement: Table S4 — Functional annotation clusters obtained with the NIH DAVID tool using differentially expressed genes in Table S3. Clusters with enrichment scores >1.5 are shown. Individual P-values listed correspond to EASE Scores, or modified Fisher Exact P-Values computed by DAVID. (DOCX) [file pone.0099625.s015.docx]

| Annotation Cluster 1 | Enrichment Score: 4.72 |  |  |
| --- | --- | --- | --- |
| Category | Term | Gene Count | P-value |
| SP_PIR_KEYWORDS | glycoprotein | 111 | 4.5E-08 |
| GOTERM_CC_FAT | GO:0031012~extracellular matrix | 23 | 1.1E-07 |
| UP_SEQ_FEATURE | glycosylation site:N-linked (GlcNAc...) | 106 | 1.7E-07 |
| GOTERM_CC_FAT | GO:0005578~proteinaceous extracellular matrix | 20 | 2.4E-06 |
| GOTERM_CC_FAT | GO:0044421~extracellular region part | 37 | 5.7E-06 |
| SP_PIR_KEYWORDS | Secreted | 52 | 6.2E-06 |
| SP_PIR_KEYWORDS | extracellular matrix | 16 | 7.8E-06 |
| SP_PIR_KEYWORDS | signal | 80 | 4.6E-05 |
| UP_SEQ_FEATURE | signal peptide | 80 | 5.8E-05 |
| GOTERM_CC_FAT | GO:0005576~extracellular region | 55 | 3.3E-04 |
| UP_SEQ_FEATURE | disulfide bond | 67 | 8.0E-04 |
| GOTERM_CC_FAT | GO:0044420~extracellular matrix part | 9 | 8.7E-04 |
| SP_PIR_KEYWORDS | disulfide bond | 68 | 1.1E-03 |
| GOTERM_CC_FAT | GO:0005604~basement membrane | 7 | 2.1E-03 |
|  |  |  |  |
| Annotation Cluster 2 | Enrichment Score: 3.38 |  |  |
| Category | Term | Gene Count | P-value |
| GOTERM_BP_FAT | GO:0001944~vasculature development | 15 | 1.5E-04 |
| GOTERM_BP_FAT | GO:0048514~blood vessel morphogenesis | 13 | 3.7E-04 |
| GOTERM_BP_FAT | GO:0001568~blood vessel development | 14 | 4.2E-04 |
| GOTERM_BP_FAT | GO:0001525~angiogenesis | 10 | 1.3E-03 |
|  |  |  |  |
| Annotation Cluster 3 | Enrichment Score: 3.15 |  |  |
| Category | Term | Gene Count | P-value |
| GOTERM_BP_FAT | GO:0003018~vascular process in circulatory system | 8 | 6.9E-05 |
| GOTERM_BP_FAT | GO:0003013~circulatory system process | 13 | 1.2E-04 |
| GOTERM_BP_FAT | GO:0008015~blood circulation | 13 | 1.2E-04 |
| GOTERM_BP_FAT | GO:0035150~regulation of tube size | 5 | 1.4E-02 |
| GOTERM_BP_FAT | GO:0050880~regulation of blood vessel size | 5 | 1.4E-02 |
|  |  |  |  |
| Annotation Cluster 4 | Enrichment Score: 3.13 |  |  |
| Category | Term | Gene Count | P-value |
| GOTERM_BP_FAT | GO:0007584~response to nutrient | 12 | 3.8E-05 |
| GOTERM_BP_FAT | GO:0031667~response to nutrient levels | 14 | 4.8E-05 |
| GOTERM_BP_FAT | GO:0009991~response to extracellular stimulus | 14 | 1.5E-04 |
| GOTERM_BP_FAT | GO:0010035~response to inorganic substance | 9 | 2.9E-02 |
| GOTERM_BP_FAT | GO:0033273~response to vitamin | 5 | 2.9E-02 |
|  |  |  |  |
| Annotation Cluster 5 | Enrichment Score: 3.13 |  |  |
| Category | Term | Gene Count | P-value |
| INTERPRO | IPR000884:Thrombospondin, type 1 repeat | 10 | 1.7E-06 |
| SMART | SM00209:TSP1 | 10 | 4.0E-06 |
| UP_SEQ_FEATURE | domain:TSP type-1 | 5 | 3.1E-04 |
| UP_SEQ_FEATURE | domain:TSP type-1 1 | 5 | 5.3E-03 |
| UP_SEQ_FEATURE | domain:TSP type-1 2 | 5 | 5.3E-03 |
| SP_PIR_KEYWORDS | heparin-binding | 5 | 2.0E-02 |
| UP_SEQ_FEATURE | domain:TSP type-1 3 | 3 | 1.1E-01 |
|  |  |  |  |
| Annotation Cluster 6 | Enrichment Score: 3.02 |  |  |
| Category | Term | Gene Count | P-value |
| GOTERM_BP_FAT | GO:0010033~response to organic substance | 31 | 1.1E-05 |
| GOTERM_BP_FAT | GO:0009725~response to hormone stimulus | 19 | 9.3E-05 |
| GOTERM_BP_FAT | GO:0009719~response to endogenous stimulus | 20 | 1.1E-04 |
| GOTERM_BP_FAT | GO:0043627~response to estrogen stimulus | 8 | 2.6E-03 |
| GOTERM_BP_FAT | GO:0042493~response to drug | 11 | 5.1E-03 |
| GOTERM_BP_FAT | GO:0048545~response to steroid hormone stimulus | 10 | 7.1E-03 |
| GOTERM_BP_FAT | GO:0032355~response to estradiol stimulus | 4 | 7.0E-02 |
|  |  |  |  |
| Annotation Cluster 7 | Enrichment Score: 2.54 |  |  |
| Category | Term | Gene Count | P-value |
| GOTERM_MF_FAT | GO:0050840~extracellular matrix binding | 5 | 1.2E-03 |
| GOTERM_BP_FAT | GO:0030198~extracellular matrix organization | 8 | 2.4E-03 |
| GOTERM_BP_FAT | GO:0043062~extracellular structure organization | 9 | 8.4E-03 |
|  |  |  |  |
| Annotation Cluster 8 | Enrichment Score: 2.42 |  |  |
| Category | Term | Gene Count | P-value |
| GOTERM_BP_FAT | GO:0048511~rhythmic process | 12 | 1.6E-05 |
| GOTERM_BP_FAT | GO:0008585~female gonad development | 8 | 1.3E-04 |
| GOTERM_BP_FAT | GO:0008406~gonad development | 10 | 1.6E-04 |
| GOTERM_BP_FAT | GO:0042698~ovulation cycle | 8 | 1.7E-04 |
| GOTERM_BP_FAT | GO:0046545~development of primary female sexual characteristics | 8 | 2.1E-04 |
| GOTERM_BP_FAT | GO:0046660~female sex differentiation | 8 | 2.1E-04 |
| GOTERM_BP_FAT | GO:0048608~reproductive structure development | 10 | 4.0E-04 |
| GOTERM_BP_FAT | GO:0045137~development of primary sexual characteristics | 10 | 4.2E-04 |
| GOTERM_BP_FAT | GO:0022602~ovulation cycle process | 7 | 7.7E-04 |
| GOTERM_BP_FAT | GO:0003006~reproductive developmental process | 14 | 7.9E-04 |
| GOTERM_BP_FAT | GO:0001542~ovulation from ovarian follicle | 4 | 1.1E-03 |
| GOTERM_BP_FAT | GO:0007548~sex differentiation | 10 | 1.4E-03 |
| GOTERM_BP_FAT | GO:0030728~ovulation | 4 | 1.7E-03 |
| GOTERM_BP_FAT | GO:0048609~reproductive process in a multicellular organism | 16 | 2.6E-02 |
| GOTERM_BP_FAT | GO:0032504~multicellular organism reproduction | 16 | 2.6E-02 |
| GOTERM_BP_FAT | GO:0007292~female gamete generation | 4 | 1.1E-01 |
| GOTERM_BP_FAT | GO:0001541~ovarian follicle development | 3 | 1.6E-01 |
| GOTERM_BP_FAT | GO:0007276~gamete generation | 9 | 3.9E-01 |
| GOTERM_BP_FAT | GO:0019953~sexual reproduction | 9 | 5.6E-01 |
| GOTERM_BP_FAT | GO:0007283~spermatogenesis | 4 | 9.1E-01 |
| GOTERM_BP_FAT | GO:0048232~male gamete generation | 4 | 9.1E-01 |
|  |  |  |  |
| Annotation Cluster 9 | Enrichment Score: 2.40 |  |  |
| Category | Term | Gene Count | P-value |
| GOTERM_MF_FAT | GO:0001871~pattern binding | 11 | 4.0E-04 |
| GOTERM_MF_FAT | GO:0030247~polysaccharide binding | 11 | 4.0E-04 |
| GOTERM_MF_FAT | GO:0005539~glycosaminoglycan binding | 9 | 3.4E-03 |
| GOTERM_MF_FAT | GO:0030246~carbohydrate binding | 14 | 1.0E-02 |
| SP_PIR_KEYWORDS | heparin-binding | 5 | 2.0E-02 |
| GOTERM_MF_FAT | GO:0008201~heparin binding | 6 | 3.6E-02 |
|  |  |  |  |
| Annotation Cluster 10 | Enrichment Score: 2.28 |  |  |
| Category | Term | Gene Count | P-value |
| GOTERM_BP_FAT | GO:0051270~regulation of cell motion | 12 | 6.4E-04 |
| GOTERM_BP_FAT | GO:0030334~regulation of cell migration | 11 | 8.4E-04 |
| GOTERM_BP_FAT | GO:0040012~regulation of locomotion | 11 | 2.2E-03 |
| GOTERM_BP_FAT | GO:0030335~positive regulation of cell migration | 6 | 2.1E-02 |
| GOTERM_BP_FAT | GO:0051272~positive regulation of cell motion | 6 | 3.0E-02 |
| GOTERM_BP_FAT | GO:0040017~positive regulation of locomotion | 6 | 3.0E-02 |
|  |  |  |  |
| Annotation Cluster 11 | Enrichment Score: 2.09 |  |  |
| Category | Term | Gene Count | P-value |
| SP_PIR_KEYWORDS | cell adhesion | 16 | 3.3E-03 |
| GOTERM_BP_FAT | GO:0022610~biological adhesion | 22 | 1.3E-02 |
| GOTERM_BP_FAT | GO:0007155~cell adhesion | 22 | 1.3E-02 |
|  |  |  |  |
| Annotation Cluster 12 | Enrichment Score: 2.07 |  |  |
| Category | Term | Gene Count | P-value |
| GOTERM_CC_FAT | GO:0044459~plasma membrane part | 57 | 9.9E-04 |
| GOTERM_CC_FAT | GO:0005887~integral to plasma membrane | 34 | 3.4E-03 |
| GOTERM_CC_FAT | GO:0031226~intrinsic to plasma membrane | 34 | 4.9E-03 |
| GOTERM_CC_FAT | GO:0005886~plasma membrane | 79 | 2.7E-02 |
| UP_SEQ_FEATURE | topological domain:Cytoplasmic | 64 | 1.0E-01 |
|  |  |  |  |
| Annotation Cluster 13 | Enrichment Score: 2.00 |  |  |
| Category | Term | Gene Count | P-value |
| UP_SEQ_FEATURE | domain:TSP type-1 | 5 | 3.1E-04 |
| INTERPRO | IPR017891:Insulin-like growth factor binding protein, N-terminal | 4 | 1.0E-03 |
| INTERPRO | IPR012395:IGFBP-related, CNN | 3 | 2.9E-03 |
| PIR_SUPERFAMILY | PIRSF036495:IGFBP_rP_CNN | 3 | 3.6E-03 |
| UP_SEQ_FEATURE | domain:IGFBP N-terminal | 4 | 3.8E-03 |
| INTERPRO | IPR000867:Insulin-like growth factor-binding protein, IGFBP | 4 | 4.7E-03 |
| PIR_SUPERFAMILY | PIRSF036495:IGFBP-related protein, CNN type | 3 | 5.3E-03 |
| SMART | SM00121:IB | 4 | 6.4E-03 |
| GOTERM_MF_FAT | GO:0005520~insulin-like growth factor binding | 4 | 9.4E-03 |
| GOTERM_MF_FAT | GO:0019838~growth factor binding | 7 | 1.1E-02 |
| INTERPRO | IPR006208:Cystine knot | 3 | 3.0E-02 |
| INTERPRO | IPR001007:von Willebrand factor, type C | 4 | 3.2E-02 |
| UP_SEQ_FEATURE | domain:VWFC | 3 | 3.3E-02 |
| SMART | SM00214:VWC | 4 | 4.2E-02 |
| UP_SEQ_FEATURE | domain:CTCK | 3 | 6.0E-02 |
| INTERPRO | IPR006207:Cystine knot, C-terminal | 3 | 6.9E-02 |
| SMART | SM00041:CT | 3 | 8.4E-02 |
|  |  |  |  |
| Annotation Cluster 14 | Enrichment Score: 1.85 |  |  |
| Category | Term | Gene Count | P-value |
| GOTERM_BP_FAT | GO:0030324~lung development | 7 | 8.2E-03 |
| GOTERM_BP_FAT | GO:0030323~respiratory tube development | 7 | 9.4E-03 |
| GOTERM_BP_FAT | GO:0060541~respiratory system development | 7 | 1.2E-02 |
| GOTERM_BP_FAT | GO:0035295~tube development | 9 | 4.2E-02 |
|  |  |  |  |
| Annotation Cluster 15 | Enrichment Score: 1.77 |  |  |
| Category | Term | Gene Count | P-value |
| GOTERM_CC_FAT | GO:0044420~extracellular matrix part | 9 | 8.7E-04 |
| GOTERM_CC_FAT | GO:0005604~basement membrane | 7 | 2.1E-03 |
| SP_PIR_KEYWORDS | basement membrane | 5 | 3.3E-03 |
| UP_SEQ_FEATURE | region of interest:Triple-helical region | 4 | 5.7E-03 |
| SP_PIR_KEYWORDS | trimer | 4 | 7.9E-03 |
| SP_PIR_KEYWORDS | triple helix | 4 | 1.3E-02 |
| SP_PIR_KEYWORDS | hydroxylysine | 4 | 1.3E-02 |
| INTERPRO | IPR008160:Collagen triple helix repeat | 6 | 1.5E-02 |
| SP_PIR_KEYWORDS | hydroxyproline | 4 | 2.1E-02 |
| GOTERM_CC_FAT | GO:0005581~collagen | 4 | 2.1E-02 |
| KEGG_PATHWAY | hsa04512:ECM-receptor interaction | 6 | 2.6E-02 |
| KEGG_PATHWAY | hsa04510:Focal adhesion | 9 | 4.8E-02 |
| UP_SEQ_FEATURE | short sequence motif:Cell attachment site | 5 | 5.3E-02 |
| SP_PIR_KEYWORDS | collagen | 5 | 6.5E-02 |
| GOTERM_MF_FAT | GO:0005201~extracellular matrix structural constituent | 5 | 6.5E-02 |
| KEGG_PATHWAY | hsa05222:Small cell lung cancer | 5 | 8.7E-02 |
| SP_PIR_KEYWORDS | hydroxylation | 4 | 1.0E-01 |
|  |  |  |  |
| Annotation Cluster 16 | Enrichment Score: 1.75 |  |  |
| Category | Term | Gene Count | P-value |
| SP_PIR_KEYWORDS | chelation | 3 | 6.6E-03 |
| UP_SEQ_FEATURE | region of interest:Beta | 3 | 1.1E-02 |
| UP_SEQ_FEATURE | region of interest:Alpha | 3 | 1.1E-02 |
| UP_SEQ_FEATURE | metal ion-binding site:Divalent metal cation; cluster A | 3 | 1.3E-02 |
| UP_SEQ_FEATURE | metal ion-binding site:Divalent metal cation; cluster B | 3 | 1.3E-02 |
| INTERPRO | IPR018064:Metallothionein, vertebrate, metal binding site | 3 | 1.5E-02 |
| SP_PIR_KEYWORDS | metal-thiolate cluster | 3 | 1.5E-02 |
| INTERPRO | IPR003019:Metallothionein superfamily, eukaryotic | 3 | 1.8E-02 |
| INTERPRO | IPR000006:Metallothionein, vertebrate | 3 | 1.8E-02 |
| PIR_SUPERFAMILY | PIRSF002564:metallothionein | 3 | 1.8E-02 |
| SP_PIR_KEYWORDS | metal binding | 3 | 3.6E-02 |
| SP_PIR_KEYWORDS | acetylated amino end | 4 | 2.1E-01 |
|  |  |  |  |
| Annotation Cluster 17 | Enrichment Score: 1.70 |  |  |
| Category | Term | Gene Count | P-value |
| GOTERM_BP_FAT | GO:0040008~regulation of growth | 16 | 1.1E-03 |
| GOTERM_BP_FAT | GO:0001558~regulation of cell growth | 11 | 2.4E-03 |
| GOTERM_BP_FAT | GO:0030308~negative regulation of cell growth | 6 | 2.3E-02 |
| GOTERM_BP_FAT | GO:0045792~negative regulation of cell size | 6 | 3.1E-02 |
| GOTERM_BP_FAT | GO:0045926~negative regulation of growth | 6 | 4.5E-02 |
| GOTERM_BP_FAT | GO:0008361~regulation of cell size | 8 | 7.3E-02 |
| GOTERM_BP_FAT | GO:0032535~regulation of cellular component size | 8 | 2.0E-01 |
|  |  |  |  |
| Annotation Cluster 18 | Enrichment Score: 1.54 |  |  |
| Category | Term | Gene Count | P-value |
| GOTERM_BP_FAT | GO:0008360~regulation of cell shape | 5 | 1.5E-02 |
| GOTERM_BP_FAT | GO:0022604~regulation of cell morphogenesis | 7 | 2.9E-02 |
| SP_PIR_KEYWORDS | cell shape | 3 | 5.5E-02 |
|  |  |  |  |
| Annotation Cluster 19 | Enrichment Score: 1.51 |  |  |
| Category | Term | Gene Count | P-value |
| GOTERM_BP_FAT | GO:0019216~regulation of lipid metabolic process | 8 | 3.7E-03 |
| GOTERM_BP_FAT | GO:0050996~positive regulation of lipid catabolic process | 3 | 2.1E-02 |
| GOTERM_BP_FAT | GO:0031329~regulation of cellular catabolic process | 5 | 2.3E-02 |
| GOTERM_BP_FAT | GO:0031331~positive regulation of cellular catabolic process | 4 | 2.5E-02 |
| GOTERM_BP_FAT | GO:0045834~positive regulation of lipid metabolic process | 4 | 4.7E-02 |
| GOTERM_BP_FAT | GO:0009896~positive regulation of catabolic process | 4 | 5.5E-02 |
| GOTERM_BP_FAT | GO:0050994~regulation of lipid catabolic process | 3 | 7.7E-02 |
| GOTERM_BP_FAT | GO:0009894~regulation of catabolic process | 5 | 9.0E-02 |
